# Author Correction: Prediction error processing and sharpening of expected information across the face-processing hierarchy

**DOI:** 10.1038/s41467-024-51267-z

**Published:** 2024-08-08

**Authors:** Annika Garlichs, Helen Blank

**Affiliations:** Department of Systems Neuroscience, University Medical Center Hamburg-Eppendorf, 20246 Hamburg, Germany

**Keywords:** Human behaviour, Sensory processing, Perception, Computational neuroscience

Correction to: *Nature Communications* 10.1038/s41467-024-47749-9, published online 22 April 2024

The original version of the Article contained an error in Figure 4c, in which the plotted means from Figure 4d were inadvertently duplicated in Figure 4c.

The article also contained an error in Figure 4j, in which for the left I/MTG, the asterisk was incorrectly placed at VGG-Face (P) instead of VGG-16 (P).

In addition, in the Results section, under the subheading ‘Prediction error and sharpened representations of expected face information in face-sensitive regions’ incorrect decimal places were reported for the mean correlation of VGG-Face (PE) in I/MTG (*M* = 0.005 corrected to *M* = 0.0005), the *p*-values of VGG-16 (PE) in pFFA (*p* = 0.002 corrected to *p* = 0.0002), and in aTL (*p* = 0.001 corrected to *p* = 0.0001). An incorrect *p*-value was reported for VGG-Face vs. VGG-16 (PE) in pFFA (*p* = 0.0212 corrected to *p* = 0.0315), and an incorrect SEMws was reported for ResNet50 (PE) in OFA (SEMws = 0.02 corrected to SEMws = 0.03).

These errors have been corrected in the PDF and HTML versions of the Article.

