# Supplementary material for: Prediction error processing and sharpening of expected information across the face-processing hierarchy
Source: Nat Commun. 2024 Apr 22;15:3407. doi: 10.1038/s41467-024-47749-9 (PMC11035707; doi:10.1038/s41467-024-47749-9)
Supplement: Supplementary file 1 — Supplementary Information [file 41467_2024_47749_MOESM1_ESM.pdf]

Supplementary Information: Prediction Error Processing and Sharpening of Expected Information Across the Face-Processing Hierarchy

Authors: Annika Garlichs[1✉] & Helen Blank[1✉]

[1]Department of Systems Neuroscience, University Medical Center Hamburg-Eppendorf,

20246 Hamburg, Germany. ✉emails: a.garlichs@uke.de, h.blank@uke.de

Supplementary Figures

S1. RSA based on the network ResNet50.

S2. Whole-brain searchlight analyses for the hypothesis models PE and Sharpening (VGG-16).

S3. Whole-brain searchlight analyses for the hypothesis models PE and Sharpening (ResNet50).

S4. Hypothesis Representational Dissimilarity Matrices (RDMs) for the object-trained neural networks VGG-16 and ResNet50.

S5. Classification analyses of the face morph images.

S6. Multidimensional scaling (MDS) (pool4) of the four face images.

S7. Calibration results of the morph selection experiment.

Supplementary Methods

M1. Image Specifications.

M2. Morph Calibration Experiment.

M3. Selection of Morph Levels in Face Pairs for Individual Participants.

M4. Training Sessions.

M5. Feedback.

M6. Behavioural Outlier.

M7. Whole-Brain Mask.

M8. Multivariate Classification Analysis.

Supplementary Results

R1. Control Indices.

R2. Reaction Time Analysis.

R3. Multivariate ROI Analyses: Left vs. Right Hemisphere.

R4. Multivariate Classification Analysis.

Supplementary Tables

T1. Univariate analysis of the contrast ‘mismatch > match’.

T2. Univariate analysis of the contrast ‘unexpected > expected’.

T3. Means and standard error of the means (SEM) of the multivariate ROI analyses (VGG-Face).

T4. P-values of the multivariate ROI analyses (VGG-Face).

T5. Means and standard error of the means (SEM) of the multivariate ROI analyses (VGG-16).

T6. P-values of the multivariate ROI analyses (VGG-16).

T7. P-values of the multivariate ROI analyses (VGG-Face vs. VGG-16).

T8. Means and standard error of the means (SEM) of the multivariate ROI analyses (ResNet50).

T9. P-values of the multivariate ROI analyses (ResNet50).

T10. Means and standard deviations for the main effects of the ROI analyses split-up by hemisphere.

T11. Main effects and post-hoc tests for the ROI analyses split-up by hemisphere (VGG-Face).

T12. Main effects and post-hoc tests for the ROI analyses split-up by hemisphere (VGG-16).

T13. Main effects and post-hoc tests for the ROI analyses split-up by hemisphere (ResNet50).

T14. Searchlight analysis for the hypothesis model PE (VGG-Face, pool4).

T15. Searchlight analysis for the hypothesis model PE (VGG-Face, pool5).

T16. Difference searchlight results for ‘PE minus Sensory’ (VGG-Face, pool4 minus conv1_2).

T17. Difference searchlight results for ‘PE minus Sensory’ (VGG-Face, pool5 minus conv1_2).

T18. Searchlight analysis for the hypothesis model Sharpening (VGG-Face, pool4).

T19. Difference searchlight results for ‘PE minus Sharpening’ (VGG-Face, pool5).

T20. Searchlight analysis for the hypothesis model Prediction Error (PE) (VGG-16, pool4).

T21. Searchlight analysis for the hypothesis model PE (VGG-16, pool5).

T22. Difference searchlight results for ‘PE minus Sensory’ (VGG-16, pool4 minus conv1_2).

T23. Difference searchlight results for ‘PE minus Sensory’ (VGG-16, pool5 minus conv1_2).

T24. Difference searchlight results for ‘PE minus Sharpening’ (VGG-16, pool4).

T25. Difference searchlight results for ‘PE minus Sharpening’ (VGG-16, pool5).

T26. Searchlight analysis for the hypothesis PE model (ResNet50, res5b_branch2b).

T27. Difference searchlight results for ‘PE minus Sensory’ (ResNet50, res5b_branch2b).

T28. Difference searchlight results for ‘PE minus Sharpening’ (ResNet50, res5b_branch2b).

T29. Localizer ‘faces > scenes’.

Supplementary Figures

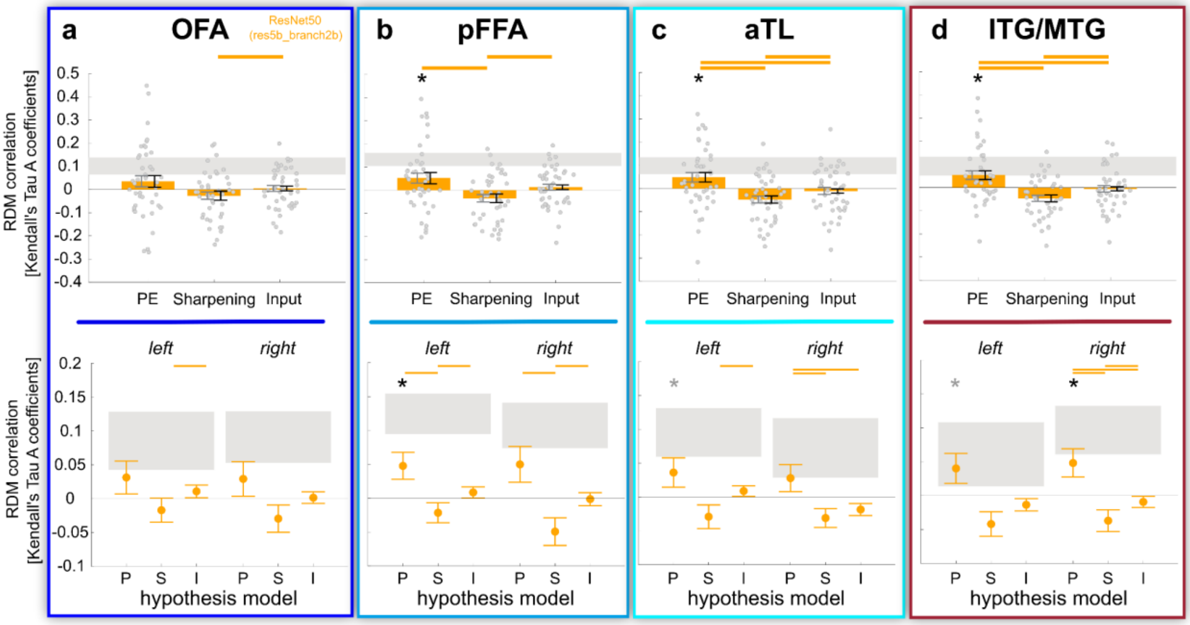

Supplementary Figure 1. RSA based on the network ResNet50[1]. a-d) We performed Representational Similarity Analysis (RSA) in our four ROIs (occipital face area, OFA; posterior fusiform face area, pFFA; anterior temporal lobe, aTL; inferior/middle temporal gyrus, ITG/MTG) and applied hypothesis models based on Prediction Error (PE), a Sharpening, and a pure Sensory Input model. For the creation of the hypothesis representational dissimilarity matrices (RDM), we extracted activations from layer res5b_branch2b in MATLAB (based on[2]). Grey bars indicate the between-subject standard error of the mean (SEM), black bars indicate the within-subject SEM[3] (*N* = 43 participants). Asterisks indicate the tests of each hypothesis model against zero (one-sided Wilcoxon signed rank test), black asterisks showing significance Bonferroni-corrected for the number of models per ROI (*N* = 3), grey asterisks showing uncorrected significance $p < .05$, and horizontal lines indicate model comparison results (two-sided paired Wilcoxon signed rank tests), FDR-corrected[4] per ROI. Grey rectangles display the lower and upper boundary of the noise ceiling for each ROI[5].

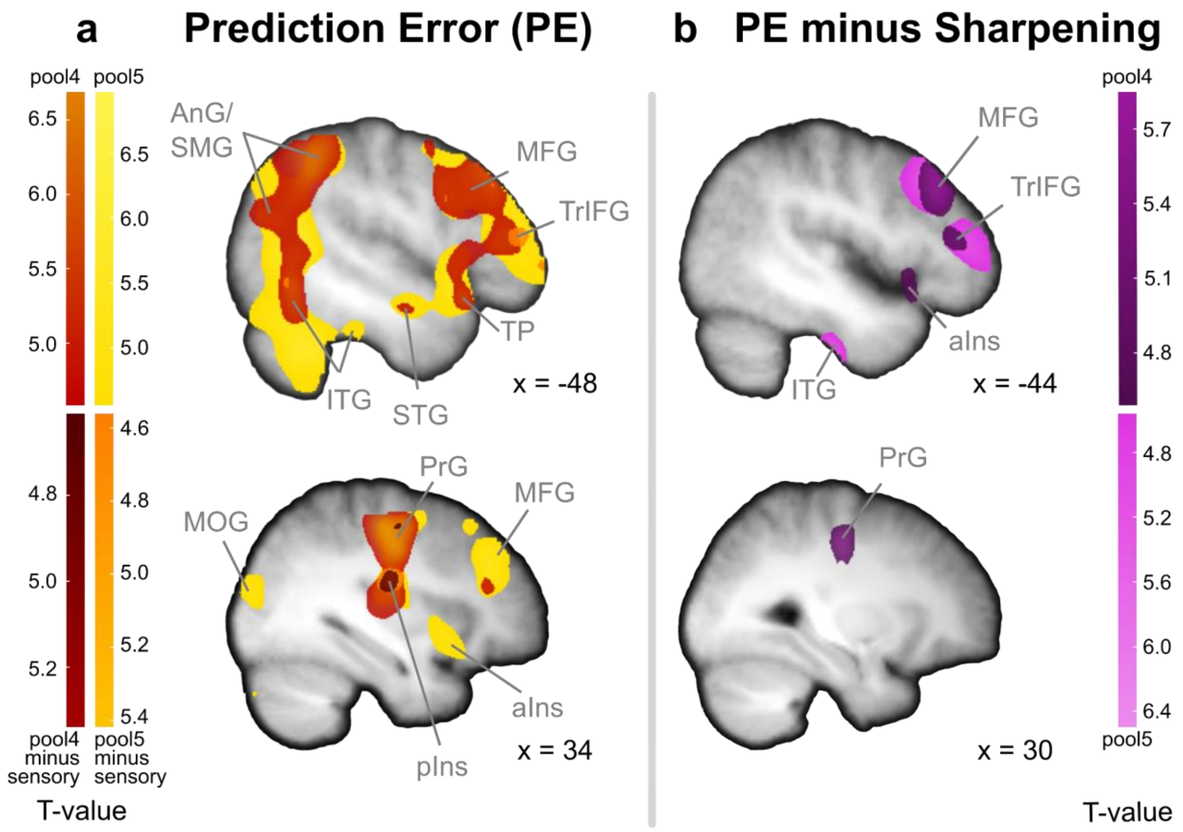

Supplementary Figure 2. Whole-brain searchlight analyses for the hypothesis models Prediction Error (PE) and Sharpening (VGG-16). Results for the comparison of the neural and hypothesised dissimilarity structures based on pool4 and pool5 layers from VGG-16 are displayed against zero and as difference maps against a sensory searchlight without prior influence based on the second convolutional layer[6], respectively. **a) Searchlight analyses results for PE**: Clusters were identified in angular gyrus (AnG), inferior occipital gyrus (IOG), triangular part of the inferior frontal gyrus (TrIFG), temporal pole (TP), superior temporal gyrus (STG), inferior temporal gyrus (ITG), middle occipital gyrus (MOG), precentral gyrus (PrG), middle frontal gyrus (MFG), anterior insula (aIns), and posterior insula (pIns). **b) Comparison of the PE > Sharpening searchlight results**: Stronger correlations for PE than Sharpening were evident in the left MFG, TrIFG, and ITG, and in the right PrG. All maps are displayed at p(FWE) < .05. Maps are overlaid on the average structural T1 image in Montreal Neurological Institute (MNI) template space.

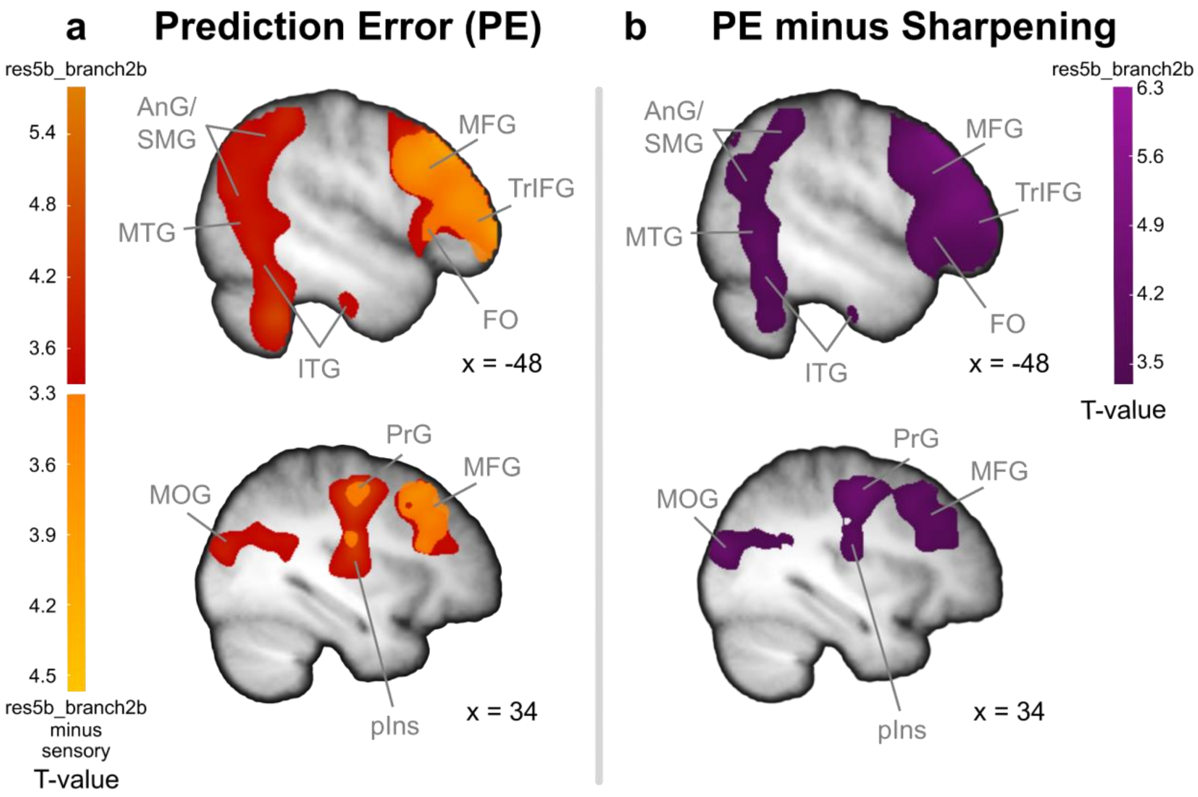

Supplementary Figure 3. Whole-brain searchlight analyses for the hypothesis models Prediction Error (PE) and Sharpening (ResNet50). Results for the comparison of the neural and hypothesised dissimilarity structures based on layer res5b_branch2b from ResNet50 are displayed against zero and as difference maps against a sensory searchlight without prior influence also based on res5b_branch2b activations. **a) Searchlight analyses results for PE**: Clusters were identified in angular gyrus (AnG), supramarginal gyrus (SMG), middle frontal gyrus (MFG), triangular part of the inferior frontal gyrus (TrIFG), frontal operculum (FO), inferior temporal gyrus (ITG), middle temporal gyrus (MTG), middle occipital gyrus (MOG), precentral gyrus (PrG), and posterior insula (pIns). **b) Comparison of the PE > Sharpening searchlight results**: Stronger correlations for PE than Sharpening were evident in the areas identified in **a)**. All maps are displayed at $p < .001$, uncorrected. Maps are overlaid on the average structural T1 image in Montreal Neurological Institute (MNI) template space.

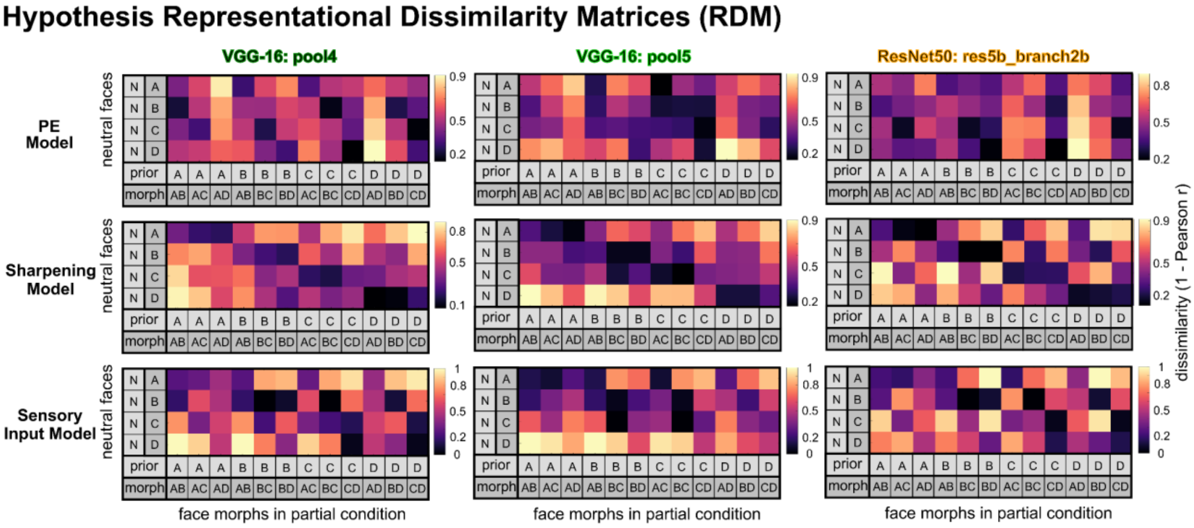

Supplementary Figure 4. Hypothesis Representational Dissimilarity Matrices (RDMs) for the object-trained neural networks VGG-16 and ResNet50. For VGG-Face, the RDMs for our hypothesis models (Prediction Error, PE; Sharpening; Sensory Input) were based on pool4 and pool5 activations. For ResNet50, the RDMs were based on activations extracted from the layer res5b_branch2b in MATLAB. For visualisation, the displayed PE and Sharpening RDMs were averaged across individual (*N* = 43) RDMs.

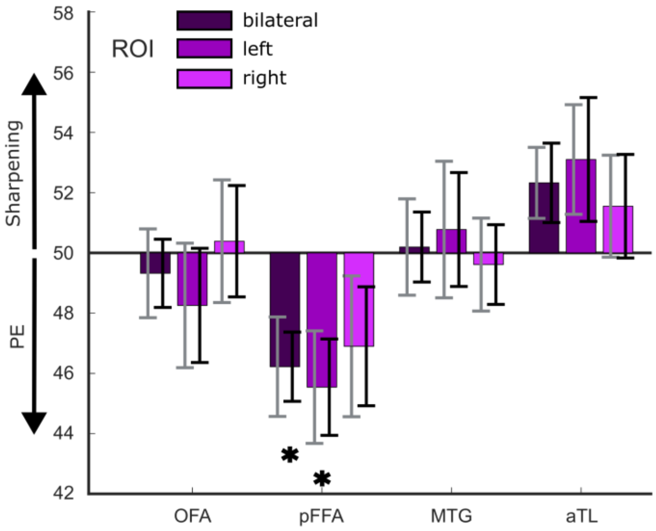

Supplementary Figure 5. Classification analyses of the face morph images. Images of face morphs were classified as either the expected or unexpected identity. Classification values above 50% indicate classification as the expected identity (i.e., Sharpening) while values below 50% indicate classification as the unexpected identity (i.e., Prediction Error (PE)). The darkest purple colour shows the mean classification values across hemispheres, the middle purple colour shows the left hemispheric values, and the light purple shows the right hemispheric values. Grey bars indicate the between-subject standard error of the mean (SEM), black bars indicate the within-subject SEM[3] (*N* = 43 participants). Asterisks show significance ($p < .05$, uncorrected). OFA = occipital face area, pFFA = posterior fusiform face area, MTG = middle temporal gyrus, aTL = anterior temporal lobe.

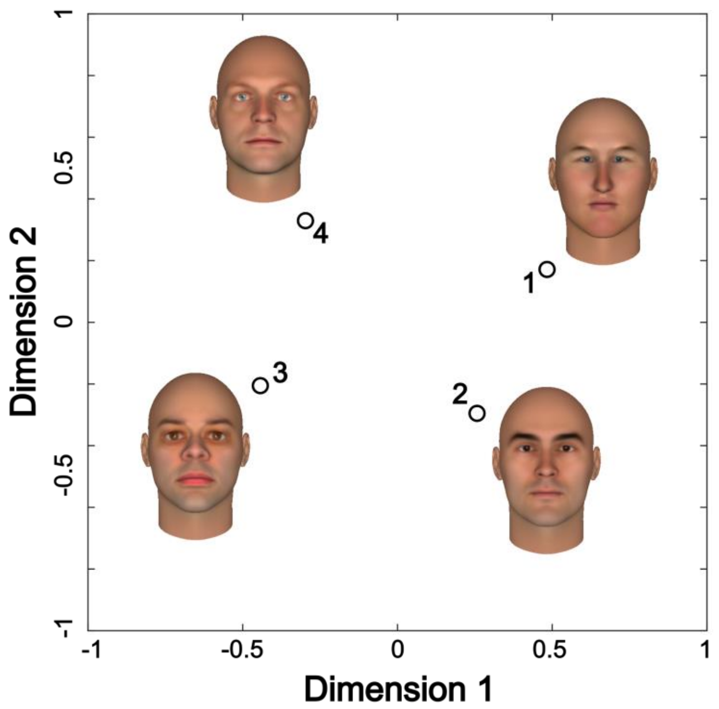

Supplementary Figure 6. Multidimensional scaling (MDS) (pool4) of the four face images. Four male identities were created using FaceGen Modeller Core 3.22. To ensure that the four selected faces were equally distinct from each other, a classical MDS was performed based on the activations of layer pool4 of the deep neural network VGG-Face[7]. The distance measure was '1 - Pearson correlation' and the dissimilarities were rescaled to values between 0 and 1. The dissimilarity structure based on two dimensions showed that the images were equally dissimilar to each other.

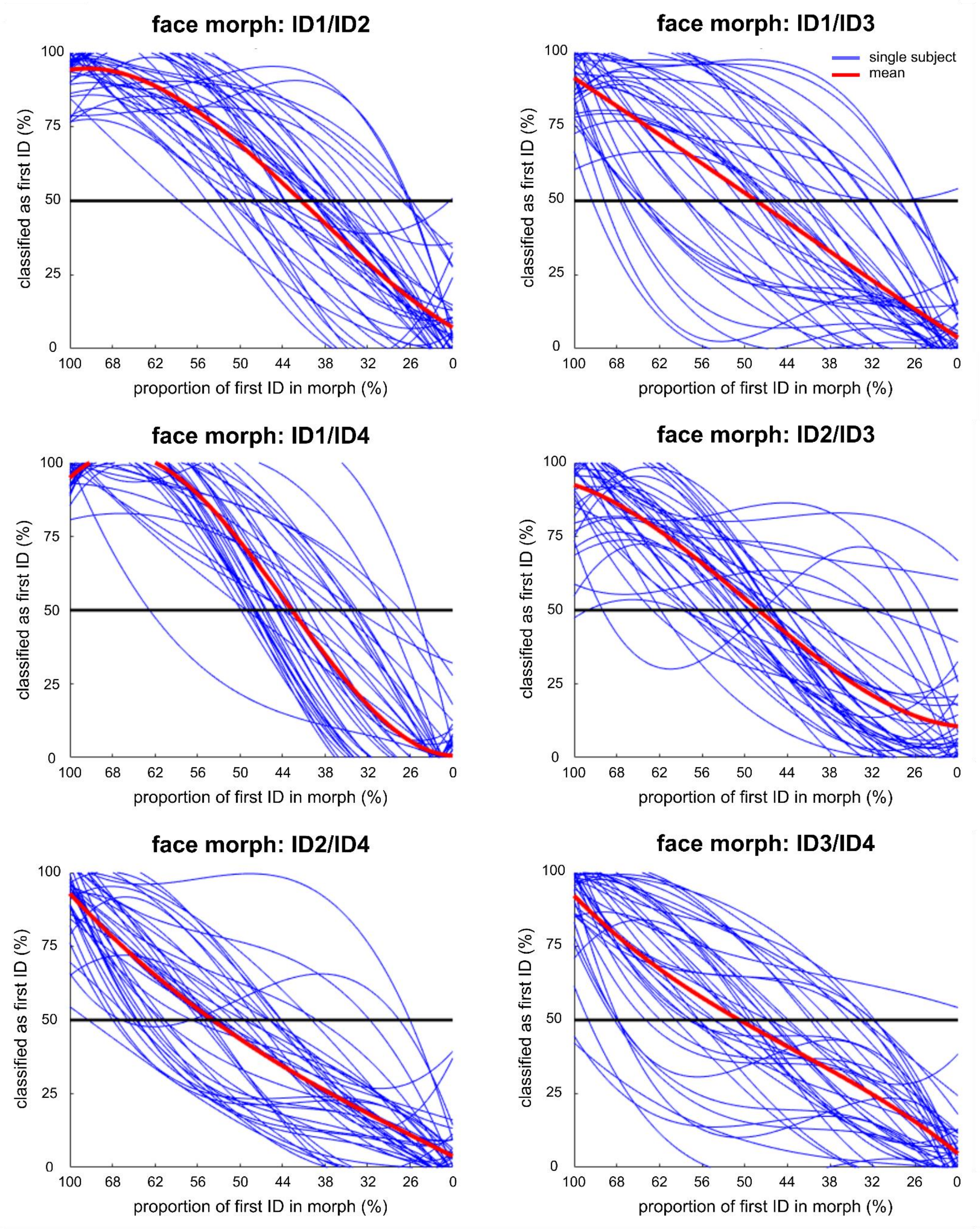

Supplementary Figure 7. Calibration results of the morph selection experiment. Participants performed a face morph calibration experiment on the day before the fMRI experiment took place (see Supplementary Methods: Morph Calibration Experiment and Selection of Morph Levels in Face Pairs for Individual Participants). The goal was to select for each participant the individual morph levels for each face pair that equaled the point of ambiguous perception (i.e., the morph level that they individually identified as the first and the

second identity in 50% of the cases). A polynomial function of 3rd order was fitted to each participant's data to grasp the typical nonlinear classification of face morphs between two identities[8]. Single participants' fitted functions are shown as blue lines (*N* = 43). The mean response function across all participants is shown in red. The black line indicates the threshold for identifying a morph level as the first and the second identity equally often, i.e., ambiguous perception.

Supplementary Methods

Image Specifications

All face images and all morphs were created in two sizes: 400 x 400 and 531 x 531 pixels. The smaller images were used for the behavioural training sessions outside of the scanner on monitors (20'') with a spatial resolution of 1920 x 1200. The distance between the retina and the monitor was approximately 72 cm. The larger images were used for the experiment in the fMRI scanner where images were displayed via a NordicNeuroLab InroomViewing Device 40'' LCD monitor with a spatial resolution of 1920 x 1080. The distance from the participant's eyes to the head coil mirror was 13 cm and from the mirror to the monitor 150 cm. The image sizes were calculated so that they had the same visual angle inside and outside of the scanner (8.59° x 8.59°).

From each original scene image, an excerpt was taken and rescaled to 531 x 531 and 400 x 400 pixels. Again, the larger images were used in the scanner for the main experiment and the functional localizer and the smaller images were used for training sessions outside of the scanner.

Morph Calibration Experiment

On the first day, participants started with the morph calibration experiment. The goal of the calibration was to identify each individual's morph level that corresponded to the 50/50 perceptual threshold at which both identities were equally often identified. For the calibration experiment, morphs between each pair of the four faces ranging from 26/74 to 68/32 (faceID1/faceID2) in steps of six percent were created. Morph windows were slightly shifted towards the less dominant face identity within each pair based on a behavioural pilot with an independent set of participants (N = 9).

In the first part of the calibration, participants learned to associate each of the four faces with their respective names. They got accustomed to the duration of 100 ms per face presentation and the additional noise applied to the faces. In the second part of the calibration, morph faces for all face pairs were presented. The task was to indicate via pressing one of four buttons with the right hand (index, middle, ring, and pinky finger) which person they saw: Ari, Bob, Cid, or Dan. If the presented face was not one of the four original faces, their task was to indicate which of the four faces they saw predominantly. Feedback was provided if the participants were too slow or if they wrongly answered a person that was not contained in a morph. The calibration was divided into three sub-blocks for five minutes: In each block, each morph level of a face pair (eight morph levels per pair) was presented twice. Additionally, each of the original faces was presented twice in each block to reinforce the originally learned face representations. This resulted in 112 trials per block. Between each block, there was a break

screen showing the original four faces and their names. The order of faces was pseudo-randomized so that the same face or face morph was consecutively presented twice at maximum. After the calibration experiment, the most ambiguous face morph for each face pair was selected (see Supplementary Methods: Selection of Morph Levels in Face Pairs for Individual Participants) and noise was added to the images for the consecutive training sessions and the main experiment.

Selection of Morph Levels in Face Pairs for Individual Participants

Based on the calibration experiment, the individual morph levels for each face pair that equaled the point of ambiguous perception were selected (see Supplementary Figure 7). We tested six morph levels per face pair. For each morph level, the number of responses in favour of the first and the second identity within the morph was counted across the three blocks of the experiment. A polynomial function of 3rd order was fitted to the data to grasp the typical nonlinear classification of face morphs between two identities[8]. Two additional morph levels (100/0 and 0/100) with perfect classification scores were added for each morph pair to obtain a better fit. The fitting of the polynomial function resulted in a vector of 1010 interpolated values representing probabilities of choosing the first identity in a face morph. All interpolated values close to 0.5 (i.e., 50% probability of identifying a morph as the first and the second identity) with a tolerance of 0.001 were extracted to identify the most ambiguous morph level. In case the polynomial function did not cross the threshold line, a morph level was chosen to counteract the perceptual dominance of one face identity in a pair. For the first three participants, the morph levels were chosen based on visual inspection of the psychometric curves.

Training Sessions

Participants came to the lab on two consecutive days to take part in the experiment. On the first day, they completed the individual face morph calibration (25 min), a training session for learning the associations between scenes and faces (25 min), and a repetition of the last part of the association training which was a short form of the final experiment (5 min). On the second day, participants started with a shortened version of the association training (15 min), followed by a short repetition of the final task (5 min). Afterwards, they completed a short part (5 min) in which the inter-trial intervals (ITI) were as long as they would be in the scanner. For all previous training sessions, ITIs were shortened to reduce overall duration. All training sessions were performed outside of the scanner. Finally, the experiment in the scanner took place (52 min) followed by a functional localizer (4 min).

After the completion of the calibration experiment (see Supplementary Methods: Morph Calibration Experiment), participants took part in an association training session in

which they learned to associate each face with a scene. The training started by having definite transition probabilities, i.e., that the faces always correctly appeared after the scene to which they belonged. The task was to correctly identify each face as Ari, Bob, Cid, or Dan. No morphs were shown during this learning phase. Later, a neutral scene was introduced after which each face was equally likely to appear. The task was still to identify the faces, however, participants should press a button with the left thumb for *whichever* face appeared after the fifth, neutral scene. The allocation of which scene was predictive of which identity and which scene was neutral was counterbalanced across participants. There were five different allocation versions. As a next step of the association training session, catch trials were introduced: In some trials, a question mark followed a scene instead of a face. If a question mark appeared, the task for the participant was to indicate which person they had anticipated after the previous scene (catch trial). If the question mark appeared after the neutral scene, they should press a button with the left index finger to signal that all faces had been equally likely to appear (neutral catch trial). Afterwards, mismatch trials were introduced. Transition probabilities for the four scenes were manipulated such that they were followed by the correct face in ~66.66 % of the trials, in ~20 % by a face which had not been anticipated, and in ~13.33 % by a question mark. In the last part, face morphs were added as partial trials. This part had the same transition probabilities and number of trials as one block in the final fMRI experiment. The part consisted of 107 trials. There were 16 match trials, 48 partial trials, 12 mismatch trials, 12 catch trials, 16 neutral trials, and 3 neutral catch trials. The transition probabilities were as follows: The four main scenes (88 trials) were followed by the expected face or a morph containing it in ~72,73 % of the trials (~18.18 % match trials, ~54.54 % partial trials)., as well as mismatch (~13.64 %) and catch trials (~13.64 %). The neutral scene (19 trials) was followed by one of the four faces in ~84.21% of the trials and neutral catch trials with a question mark (~15.79 %). An exemplary trial can be seen in Figure 1b.

For the last part of the association training, participants were given feedback (see Supplementary Methods: Feedback) by the experimental leader when there were misunderstandings concerning the task and brief feedback about single mistakes was also provided if participants performed well. Finally, participants repeated the last part of the association training session and received a second feedback with improvements or still occurring errors. Overall, participants had learned and understood the task (accuracy: M = 85.50 %, SD = 7.54 %).

On the second day, participants completed a shortened version of the association training session and obtained feedback. The task and condition distribution of the final part was identical to one block in the fMRI experiment. The participants were informed that the timing in the scanner was different due to the specificities of the MRI scanner. They completed a four-minute experiment with longer ITIs (1500-2500 ms, mean: 2000 ms) and null events

(fixation cross) with a trial duration of 5300 ms so that they would not be surprised in the scanner.

Feedback

Participants received verbal feedback after completion of the training sessions and after each block of the fMRI experiment. They were given an overall score of their performance which was the mean of the accuracy scores for the match, partial, mismatch, catch, neutral, and neutral catch trials, as well as individual feedback for the different conditions. Partial trials were classified as correct if the participant answered in time to have perceived one of the two identities that were present in the morph. Feedback for specific conditions was provided if participants had not understood the task.

Behavioural Outlier

Although in the preregistration we had not defined an exclusion criterion, we decided to exclude one participant who performed worse than 99.9% of the participants (i.e., z = -4.16 for the z-standardised mean accuracy based on the match, mismatch, partial, catch, neutral, and neutral catch conditions). Therefore, the reported results are based on 43 participants.

Whole-Brain Mask

For the first-level analyses of the main experiment and the functional localizer, individual whole-brain masks were created based on grey matter, white matter, and cerebrospinal fluid probability maps obtained from SPM12's segmentation-function. The threshold for all three tissue types was set to 0. The mask was additionally smoothed with a 2-mm full-width at half maximum isotropic Gaussian kernel and its resulting mask binarized using a threshold of 0. For the second-level analysis, a group-level whole-brain mask was created by averaging the individual skull-stripped normalised anatomies. The average anatomy was then thresholded at 0.6, smoothed at 8-mm full-width, and finally binarized.

Anatomical labels of brain regions reported in the Supplementary Tables were taken from the Neuromorphometrics atlas (Neuromorphometrics, Inc.) implemented in SPM12 and the Harvard-Oxford Cortical and Subcortical Structural Atlases implemented in FSLeyes. In the case of white matter localizations, the respective brain lobe was provided for anatomical orientation.

Multivariate Classification Analysis

In addition to the RSA, we conducted a simpler multivariate classification approach without model-based hypotheses RDMs by using The Decoding Toolbox[9]. We used L2-norm support vector machines (SVM) from the library LIBSVM[10] and performed a classification

analysis. To answer the question whether a 50/50 morphed face is classified as the expected or unexpected face identity based on voxel-based activation patterns, we trained individual classifiers on the T-images of pairs of the neutral faces and tested them for each corresponding morph combination (AB, AC, AD, BC, BD, CD) across the four runs. The classifier's performance, i.e., classification score, was evaluated using classification accuracy minus chance (50%). We labelled the T-images of the test set so that classification scores larger than .5 indicate that a morph was classified as the expected face and classification scores below .5 indicate that a morph was classified as the unexpected face. Classification analyses were separately conducted for each participant, for each morph combination and each ROI separated by hemisphere and averaged across these to obtain a total classification score per participant per ROI. As classification scores were not normally distributed (Kolmogorov-Smirnow tests, all $p < .001$), we used two-sided Wilcoxon signed rank tests for all classification analyses.

Supplementary Results

Control Indices

To control whether participants were attentive and correctly performed the task, a Friedman test with the within-subject factor condition (match, mismatch, catch, neutral, neutral catch) and accuracy (%) as the dependent variable was calculated. We performed a non-parametric test instead of the preregistered ANOVA because the accuracies were not normally distributed due to ceiling effects (Kolmogorow-Smirnow tests, all $p < .001$). Match and mismatch trials were classified as correct if the presented (unmorphed) face was correctly identified in time. In catch trials, participants had to correctly answer the question mark by indicating which person they expected based on the preceding scene. Neutral trials were correct if participants pressed the button with the left thumb irrespective of which face was presented and neutral catch trials were correctly answered if they pressed the button with the left index finger to indicate that all persons were equally likely to be expected. Note that for the conditions mentioned, no morphs were presented.

Participants performed well in all experimental control conditions, especially in the match condition. There was a significant difference in accuracy ($\chi^2(4) = 32.17$, $p < .001$, Kendall's W = 0.19). Tukey's HSD test for multiple comparisons showed that participants significantly more often correctly identified a face in the match (*M* = 97.64%, *SD* = 3.98%) compared to the mismatch (*M* = 90.94, *SD* = 11.77, $p < .001$, LB/UB [0.62, 2.33]), catch (*M* = 95.11, *SD* = 5.12, $p = .049$, [0.003, 1.72]), and neutral catch condition (*M* = 91.28, *SD* = 9.08;

$p < .001$, [0.44, 2.16]). Participants also showed higher accuracy in the neutral compared to the neutral catch condition ($p = .01$, [0.13, 1.85]), possibly due to the more frequent occurrence of neutral compared to neutral catch trials. No difference in accuracy was observed between the match and neutral condition ($M = 96.22$, $SD = 5.48$; $p = .86$, [-0.54, 1.17]) nor in any other condition comparison.

Reaction Time Analysis

A Friedman test with the within-subject factor condition (match, mismatch, partial, neutral) and RT as the dependent variable was conducted. Additionally to the results reported in the main text, in the neutral condition, participants responded faster ($M = 547.7$, $SD = 55.84$) compared to all other conditions (match: $p = .047$, LB/UB [0.01, 1.44]; mismatch: $p < .001$, [1.70, 3.13]; partial: $p < .001$, [-3.02, -1.59]), because they simply had to press a fifth button for whichever face appeared after the neutral scene.

Multivariate ROI Analyses: Left vs. Right Hemisphere

In our main multivariate ROI analyses, we investigated the correlations between the hypothesis models and bilateral ROIs along the ventral face-processing hierarchy. We performed additional analyses to test whether the correlations differed between the left and the right hemispheres (see Figure 4g-j, Supplementary Figure 1). We tested for main effects and interactions between the factors hemisphere (left, right) and model (PE, Sharpening, Sensory Input) for each ROI and each deep neural network with the non-parametric Aligned Rank Transform (ART)[11] and calculated η_p^2 as a measure of effect size.

There was no significant main effect of hemisphere for any of the three neural networks (VGG-Face, VGG-16, ResNet50) in any of the ROIs (OFA, pFFA, aTL, MTG), i.e., the correlations of the neural data with the three hypothesis models (PE, Sharpening, Sensory Input) did not differ between the left and right hemisphere (all p(unc.) < .05; see Supplementary Tables 10-13).

There was a significant main effect of model for every neural network in every ROI (all p(unc.) < .05; see Supplementary Tables 10-13). For VGG-Face, post-hoc pairwise comparisons[12], Tukey-corrected, revealed that these main effects were mainly driven by higher correlation values with the PE model compared to the Sensory Input model in OFA and pFFA as well as higher correlation values for the PE model compared to the Sharpening model in pFFA, aTL, and ITG/MTG (see Supplementary Table 10, 11). The object-trained network VGG-16 showed a similar pattern, with stronger correlations of the PE model compared to the Sensory Input model in pFFA and aTL as well as stronger PE correlations compared to Sharpening correlations in all ROIs (see Supplementary Tables 10, 12). Additionally, there were stronger correlations of the Sensory Input model compared to the Sharpening model in

pFFA. The neural network ResNet50 showed similar higher correlations for the PE model, mainly compared to the Sharpening model in all ROIs, but contrary to the other object-trained network VGG-16, it did not reveal any evidence for stronger correlations with the Sensory Input model compared to the other hypothesis models (see Supplementary Tables 10, 13).

Multivariate Classification Analysis

In the classification analysis, ambiguous morphed faces were more often classified as the unexpected face identity based on multivariate activation patterns in the posterior FFA (M = 46.22, SD = 10.83, z = -2.17, p = .03), especially in the left FFA (M = 45.54, SD = 12.25, z = -2.29, p = .02; see Supplementary Figure 5). Classification as the expected or unexpected face did not differ in OFA (M = 49.32, SD = 9.66, p = .72, z = -0.35), aTL (M = 52.33, SD = 7.73, p = .055, z = 1.92), and MTG (M = 50.19, SD = 10.48, p = .93, z = 0.09). Hence, the classification analysis confirms the RSA results for PE representations in the FFA, while classification did not reveal either PE or Sharpening in the other ROIs. Overall, both multivariate analyses approaches, i.e., RSA and classification analysis, suggest concurrent PE and sharpened face representations. The major difference between the two different multivariate analyses approaches is that RSA allows us to test for both PE and sharpened face representations simultaneously in one region, while the classification approach is forced to classify morphed faces as either the expected or unexpected face. In addition, the RSA approach also takes the similarity of a morphed face with all neutral faces into account (see hypothesis RDMs in Figure 2b and Supplementary Figure 4, which are not empty in the off-diagonal), while the classification approach only compares a morphed face with the two corresponding neutral face images. Furthermore, the classification analysis we implemented here was a model-free approach. In contrast, our RSA approach relied on hypothesis RDMs generated from various DCNN layer activations, derived from images based on combinations of expected and presented face images with PE or sharpening computations.

Supplementary Tables

Supplementary Table 1. Univariate analysis of the contrast 'mismatch > match'.

cluster *p*(FWE)	cluster equivk	peak *p*(FWE)	peak T	peak equivZ	x,y,z {mm}	label
0.000	7236	0.000	11.04	7.52	-36 -42 48	left superior parietal lobule
		0.000	9.93	7.08	42 -32 48	right supramarginal gyrus
		0.000	9.65	6.97	-44 -32 48	
0.000	6702	0.000	10.87	7.46	-4 12 50	left supplementary motor cortex
		0.000	10.58	7.34	-30 24 -6	left anterior insula
		0.000	10.42	7.29	2 20 46	
0.000	3052	0.000	10.35	7.25	32 26 -4	right anterior insula
		0.000	9.87	7.06	34 24 4	
		0.009	6.15	5.16	50 8 38	
0.000	716	0.002	6.72	5.51	38 -52 -32	right cerebellum exterior
		0.010	6.11	5.14	26 -52 -26	
		0.014	5.99	5.06	30 -62 -30	
0.000	1755	0.003	6.49	5.37	-6 -16 12	left thalamus proper
		0.012	6.06	5.11	10 10 2	right caudate
		0.016	5.95	5.04	-8 -16 -12	
0.005	309	0.010	6.12	5.15	32 -74 -52	right cerebellum exterior
		0.992	3.74	3.46	36 -64 -42	
0.000	647	0.012	6.04	5.09	12 -76 -24	right cerebellum exterior
		0.041	5.61	4.82	-8 -78 -30	
		0.098	5.28	4.60	8 -82 -30	
0.015	246	0.054	5.50	4.75	-12 -56 -46	left cerebellum exterior
		0.383	4.70	4.19	-12 -54 -38	
		0.412	4.66	4.16	-2 -54 -38	
0.000	499	0.088	5.32	4.63	16 -60 -48	right cerebellum exterior
		0.092	5.30	4.61	28 -54 -54	
		0.469	4.59	4.11	16 -66 -60	
0.005	312	0.104	5.25	4.58	-56 -42 -18	left inferior temporal gyrus
		0.856	4.14	3.77	-68 -38 -16	

0.000	479	0.115	5.21	4.55	-38 -56 -32	left cerebellum exterior
		0.210	4.97	4.38	-36 -68 -28	
		0.310	4.80	4.26	-28 -54 -32	
0.001	403	0.136	5.15	4.51	22 54 -14	right anterior orbital gyrus
		0.932	4.00	3.66	42 48 -16	
		0.976	3.86	3.55	34 50 -18	

Note. For the univariate contrast ‘mismatch > match’, a one-sample *t*-test was performed ($N = 43$ participants). Clusters at p(FWE) > .05, with a cluster-inducing threshold of p(unc.) < .001, are reported ($k > 10$ voxels).

Supplementary Table 2. Univariate analysis of the contrast ‘unexpected > expected’.

cluster *p*(FWE)	cluster equivk	peak *p*(FWE)	peak T	peak equivZ	x,y,z {mm}	label
0.000	13699	0.000	9.49	6.90	-36 -36 44	left superior parietal lobule
		0.000	8.71	6.55	20 -52 -22	
		0.000	8.35	6.38	-8 -62 46	
0.000	1829	0.000	7.73	6.07	40 -44 48	right superior parietal lobule
		0.000	7.27	5.82	40 -34 48	
		0.190	4.97	4.39	38 -56 46	
0.000	4469	0.000	7.28	5.82	26 -6 52	right superior frontal gyrus
		0.001	6.93	5.63	-24 -6 52	
		0.002	6.61	5.44	-4 36 24	left anterior cingulate gyrus
0.000	5723	0.001	6.91	5.62	10 -6 8	right thalamus proper
		0.003	6.50	5.38	10 -14 6	
		0.003	6.49	5.37	34 20 -2	right anterior insula
0.000	2271	0.029	5.70	4.88	-42 48 16	left middle frontal gyrus
		0.033	5.65	4.85	-20 50 -18	
		0.060	5.43	4.70	-28 54 -12	left anterior orbital gyrus
0.000	635	0.092	5.26	4.59	30 46 -18	right anterior orbital gyrus
		0.150	5.07	4.45	34 60 -10	
		0.463	4.56	4.09	38 44 -4	
0.001	444	0.197	4.96	4.37	40 36 20	right frontal pole
		0.336	4.72	4.21	44 34 30	right middle frontal gyrus
		0.781	4.20	3.82	46 36 38	
0.031	223	0.214	4.92	4.35	54 -32 -12	right middle temporal gyrus
		0.227	4.90	4.33	56 -40 -14	
0.004	348	0.510	4.51	4.05	-56 -40 -14	left middle temporal gyrus
		0.706	4.29	3.88	-54 -26 -26	
		0.991	3.71	3.43	-58 -18 -30	

Note. For the univariate contrast ‘unexpected > expected’, a one-sample *t*-test was performed ($N = 43$ participants). Clusters at *p*(FWE) > .05, with a cluster-inducing threshold of *p*(unc.) < .001, are reported ($k > 10$ voxels).

Supplementary Table 3. Means and standard error of the means (SEM) of the multivariate ROI analyses (VGG-Face).

	PE			Sharpening			Input		
**ROI**	**mean**	**SEM (bw)**	**SEM (ws)**	**mean**	**SEM (bw)**	**SEM (ws)**	**mean**	**SEM (bw)**	**SEM (ws)**
**bilateral**									
**OFA**	0.0553	0.0178	0.0136	0.0283	0.0131	0.0110	0.0096	0.0133	0.0087
**pFFA**	0.0408	0.0158	0.0133	-0.0108	0.0138	0.0173	-0.0006	0.0121	0.0101
**aTL**	0.0341	0.0195	0.0144	-0.0172	0.0141	0.0150	-0.0131	0.0149	0.0120
**ITG/ MTG**	0.0418	0.0177	0.0167	-0.0074	0.0141	0.0142	0.0005	0.0145	0.0116
**unilateral**									
**lOFA**	0.0552	0.0172	0.0131	0.0236	0.0134	0.0103	0.0196	0.0148	0.0095
**rOFA**	0.0517	0.0178	0.0135	0.0241	0.0128	0.0112	0.0062	0.0115	0.0075
**lpFFA**	0.0426	0.0133	0.0131	-0.0213	0.0134	0.0158	-0.0016	0.0117	0.0096
**rpFFA**	0.0240	0.0197	0.0166	-0.0182	0.0146	0.0176	-0.0136	0.0136	0.0117
**laTL**	0.0481	0.0188	0.0139	-0.0086	0.0153	0.0154	0.0068	0.0165	0.0114
**raTL**	0.0146	0.0170	0.0156	-0.0053	0.0129	0.0140	-0.0123	0.0114	0.0101
**lITG/ MTG**	0.0212	0.0165	0.0156	-0.0067	0.0160	0.0169	-0.0080	0.0158	0.0112
**rITG/ MTG**	0.0353	0.0177	0.0151	-0.009	0.0134	0.0154	0.0002	0.0131	0.0112

Note. This table provides the means and SEMs (N = 43) for the DCNN VGG-Face in Figure 4. Individual correlation coefficients (Kendall's Tau A) between the representational dissimilarity matrices (RDM) of the three hypothesis models (PE, Sharpening, Sensory Input) and the neural RDM were calculated. Correlations for the occipital face area (OFA) are based on pool4 activations, the correlations for the other ROIs on pool5 activations. Within-subject (ws) SEMs were calculated considering the individuals' correlation coefficients across the two compared DCNNs (VGG-Face and VGG-16). For p-values of the model tests against zero and the model comparison tests, please refer to the Supplementary Table 4. pFFA = posterior fusiform face area; aTL = anterior temporal lobe; ITG = inferior temporal gyrus; MTG = middle temporal gyrus; bw = between-subject; ws = within-subject.

Supplementary Table 4. P-values of the multivariate ROI analyses (VGG-Face).

ROI	PE vs. 0	Sharpening vs. 0	Input vs. 0	PE vs. Sharpening	PE vs. Input	Sharpening vs. Input
**bilateral**						
**OFA**	0.0030*	0.0232*	0.2913	0.2941	0.0396*	0.2941
**pFFA**	0.0044*	0.5528	0.4140	0.0315*	0.0315*	0.6311
**aTL**	0.0780	0.8462	0.7838	0.0460*	0.0154*	0.7766
**ITG/ MTG**	0.0168*	0.8641	0.5457	0.0366*	0.0366*	0.8468
**unilateral**						
**lOFA**	0.0013*	0.0319*	0.0961	0.1624	0.1624	0.7766
**rOFA**	0.0048*	0.0321*	0.3451	0.3042	0.0424*	0.3917
**lpFFA**	0.0016*	0.8729	0.5866	0.0044*	0.0192*	0.2004
**rpFFA**	0.2381	0.8841	0.8242	0.1460	0.0778	0.9278
**laTL**	0.0206*	0.7820	0.3145	0.0202*	0.0202*	0.2746
**raTL**	0.2322	0.6482	0.9432	0.4373	0.2529	0.9615
**lITG/ MTG**	0.1256	0.7620	0.7542	0.3443	0.3443	0.5232
**rITG/ MTG**	0.0263*	0.8847	0.4726	0.0928	0.0928	0.4826

Note. This table reports the precise *p*-values for the DCNN VGG-Face in Figure 4 and corresponds to the data in Supplementary Table 3. We tested for the significance of the hypothesis model correlations against a null correlation using one-sided Wilcoxon signed-rank tests. Model comparisons were tested using paired, two-sided Wilcoxon signed rank tests. For the tests against zero, a black asterisk indicates Bonferroni-corrected significance considering the number of tests per ROI (N = 6 (3 models x 2 DCNNs); p < .0083), a grey asterisk indicates uncorrected significance (p(unc.) < .001). For the model comparisons, an asterisk indicates FDR-corrected[4] significance for the model comparisons per ROI. OFA = occipital face area; pFFA = posterior fusiform face area aTL = anterior temporal lobe; ITG = inferior temporal gyrus; MTG = middle temporal gyrus.

Supplementary Table 5. Means and standard error of the means (SEM) of the multivariate ROI analyses (VGG-16).

	PE			Sharpening			Input		
ROI	mean	SEM (bw)	SEM (ws)	mean	SEM (bw)	SEM (ws)	mean	SEM (bw)	SEM (ws)
**bilateral**									
**OFA**	0.0623	0.0191	0.0173	-0.0146	0.019	0.0216	0.0206	0.0142	0.0091
**pFFA**	0.101	0.0245	0.0223	-0.0099	0.0197	0.0193	0.0413	0.0207	0.0154
**aTL**	0.0963	0.0219	0.0205	-0.0396	0.0153	0.0163	0.0272	0.0183	0.0135
**ITG/ MTG**	0.0760	0.0225	0.0209	-0.0127	0.0193	0.0188	0.0357	0.0211	0.0144
**unilateral**									
**lOFA**	0.0523	0.0179	0.0161	-0.0124	0.0172	0.0191	0.0284	0.0147	0.0087
**rOFA**	0.0535	0.0202	0.0178	-0.0076	0.0186	0.0223	0.0172	0.0137	0.0096
**lpFFA**	0.0971	0.0233	0.0212	-0.0055	0.0181	0.0172	0.0460	0.0223	0.0159
**rpFFA**	0.0843	0.0261	0.0235	-0.0205	0.0197	0.0206	0.0212	0.0208	0.0152
**laTL**	0.0831	0.0206	0.0194	-0.0266	0.0163	0.0186	0.0254	0.0176	0.0129
**raTL**	0.0572	0.0176	0.0163	-0.0162	0.0175	0.0147	0.0236	0.0202	0.0143
**lITG/ MTG**	0.0531	0.0248	0.0243	-0.0146	0.0212	0.0203	0.0153	0.0222	0.0151
**rITG/ MTG**	0.0662	0.0226	0.0207	-0.0037	0.0181	0.0187	0.0373	0.0198	0.0137

Note. This table provides the means and SEMs (N = 43) for the DCNN VGG-16 in Figure 4. Individual correlation coefficients (Kendall's Tau A) between the representational dissimilarity matrices (RDM) of the three hypothesis models (PE, Sharpening, Sensory Input) and the neural RDM were calculated. Correlations for the occipital face area (OFA) are based on pool4 activations, the correlations for the other ROIs on pool5 activations. Within-subject (ws) SEMs were calculated considering the individuals' correlation coefficients across the two compared DCNNs (VGG-Face and VGG-16). For *p*-values of the model tests against zero and the model comparison tests, please refer to the Supplementary Table 6. pFFA = posterior fusiform face area; aTL = anterior temporal lobe; ITG = inferior temporal gyrus; MTG = middle temporal gyrus; bw = between-subject; ws = within-subject.

Supplementary Table 6. P-values of the multivariate ROI analyses (VGG-16).

ROI	PE vs. 0	Sharpening vs. 0	Input vs. 0	PE vs. Sharpening	PE vs. Input	Sharpening vs. Input
**bilateral**						
**OFA**	0.0025*	0.7045	0.0611	0.1351	0.1471	0.1471
**pFFA**	0.0002*	0.6322	0.0379*	0.0086*	0.0315*	0.0433*
**aTL**	0.0001*	0.9871	0.0901	0.0010*	0.0047*	0.0026*
**ITG/ MTG**	0.0038*	0.6227	0.0797	0.0366*	0.1922	0.0366*
**unilateral**						
**lOFA**	0.0032*	0.8093	0.0374*	0.1410	0.3881	0.1379
**rOFA**	0.0099*	0.5096	0.1148	0.3339	0.3042	0.4826
**lpFFA**	0.0002*	0.6637	0.0249*	0.0050*	0.1206	0.0244*
**rpFFA**	0.0018*	0.7270	0.1509	0.0395*	0.0395*	0.2551
**laTL**	0.0003*	0.9221	0.0428*	0.0092*	0.0202*	0.0202*
**raTL**	0.0017*	0.8177	0.1956	0.0313*	0.1575	0.0630
**lITG/ MTG**	0.0206*	0.7506	0.3451	0.3443	0.3443	0.3443
**rITG/ MTG**	0.0051*	0.6459	0.0411*	0.0928	0.4066	0.0928

Note. This table reports the precise *p*-values for the DCNN VGG-16 in Figure 4 and corresponds to the data in Supplementary Table 5. We tested for the significance of the hypothesis model correlations against a null correlation using one-sided Wilcoxon signed-rank tests. Model comparisons were tested using paired, two-sided Wilcoxon signed rank tests. For the tests against zero, a black asterisk indicates Bonferroni-corrected significance considering the number of tests per ROI (*N* = 6 (3 models x 2 DCNNs); $p < .0083$), a grey asterisk indicates uncorrected significance (*p*(unc.) < .001). For the model comparisons, an asterisk indicates FDR-corrected[4] significance for the model comparisons per ROI. OFA = occipital face area; pFFA = posterior fusiform face area aTL = anterior temporal lobe; ITG = inferior temporal gyrus; MTG = middle temporal gyrus.

Supplementary Table 7. P-values of the multivariate ROI analyses (VGG-Face vs. VGG-16).

ROI	PE vs. PE	Sharpening vs. Sharpening	Input vs. Input
**bilateral**			
**OFA**	0.9134	0.0396*	0.1471
**pFFA**	0.0315*	0.8185	0.0715
**aTL**	0.0176*	0.2970	0.0837
**ITG/MTG**	0.3105	0.8468	0.1212
**unilateral**			
**lOFA**	0.6743	0.1379	0.1624
**rOFA**	0.6799	0.3042	0.2506
**lpFFA**	0.0192*	0.3979	0.0192*
**rpFFA**	0.0778	0.9278	0.2178
**laTL**	0.1247	0.4762	0.3965
**raTL**	0.1556	0.9247	0.1575
**lITG/ MTG**	0.3702	0.8232	0.3443
**rITG/ MTG**	0.4066	0.6726	0.1029

Note. This table reports the precise *p*-values for the model comparisons of the DCNNs VGG-Face and VGG-16 in Figure 4 and corresponds to the data in Supplementary Table 3 and 5. An asterisk indicates FDR-corrected[4] significance for the model comparisons per ROI. OFA = occipital face area; pFFA = posterior fusiform face area aTL = anterior temporal lobe; ITG = inferior temporal gyrus; MTG = middle temporal gyrus.

Supplementary Table 8. Means and standard error of the means (SEM) of the multivariate ROI analyses (ResNet50).

	PE			Sharpening			Input		
ROI	mean	SEM (bw)	SEM (ws)	mean	SEM (bw)	SEM (ws)	mean	SEM (bw)	SEM (ws)
**bilateral**									
**OFA**	0.0355	0.0240	0.0253	-0.0264	0.0156	0.0196	0.0048	0.0132	0.0098
**pFFA**	0.0521	0.0214	0.0247	-0.0347	0.0156	0.0184	0.0131	0.0130	0.0098
**aTL**	0.0484	0.0204	0.0207	-0.0475	0.0146	0.0155	-0.0107	0.0153	0.0093
**ITG/ MTG**	0.0542	0.0187	0.0189	-0.0465	0.0133	0.0147	-0.0053	0.0143	0.0085
**unilateral**									
**lOFA**	0.0313	0.0221	0.0242	-0.0171	0.0160	0.0177	0.0107	0.0136	0.0095
**rOFA**	0.0292	0.0256	0.0255	-0.0295	0.0148	0.0203	0.0014	0.0123	0.0086
**lpFFA**	0.0478	0.0181	0.0199	-0.0213	0.0131	0.0148	0.0087	0.0120	0.0083
**rpFFA**	0.0500	0.0236	0.0263	-0.0491	0.0170	0.0204	-0.0012	0.0135	0.0096
**laTL**	0.0363	0.0218	0.0217	-0.0290	0.0161	0.0175	0.0089	0.0135	0.0077
**raTL**	0.0281	0.0181	0.0201	-0.0309	0.0138	0.0140	-0.0183	0.0140	0.0088
**lITG/ MTG**	0.0401	0.0196	0.0223	-0.0419	0.0164	0.0180	-0.0135	0.0146	0.0089
**rITG/ MTG**	0.0482	0.0197	0.0208	-0.0370	0.0148	0.0159	-0.0093	0.0137	0.0082

Note. This table provides the means and SEMs (N = 43) for the DCNN ResNet50 in Supplementary Figure 1. Individual correlation coefficients (Kendall's Tau A) between the representational dissimilarity matrices (RDM) of the three hypothesis models (PE, Sharpening, Sensory Input) and the neural RDM were calculated. Correlations were based on res5b_branch2b activations. For *p*-values of the model tests against zero and the model comparison tests, please refer to the Supplementary Table 9. OFA = occipital face area; pFFA = posterior fusiform face area; aTL = anterior temporal lobe; ITG = inferior temporal gyrus; MTG = middle temporal gyrus; bw = between-subject; ws = within-subject.

Supplementary Table 9. P-values of the multivariate ROI analyses (ResNet50).

ROI	PE vs. 0	Sharpening vs. 0	Input vs. 0	PE vs. Sharpening	PE vs. Input	Sharpening vs. Input
**bilateral**						
**OFA**	0.1011	0.9522	0.3399	0.1726	0.3310	0.0420*
**pFFA**	0.0155*	0.9781	0.1171	0.0327*	0.1359	0.0038*
**aTL**	0.0151*	0.9981	0.7132	0.0109*	0.0204*	0.0110*
**ITG/ MTG**	0.0064*	0.9988	0.7350	0.0014*	0.0062*	0.0014*
**unilateral**						
**lOFA**	0.1003	0.8547	0.1716	0.3008	0.6420	0.0203*
**rOFA**	0.1625	0.9659	0.3496	0.2502	0.4116	0.1255
**lpFFA**	0.0111*	0.9506	0.2381	0.0342*	0.0902	0.0284*
**rpFFA**	0.0503	0.9935	0.4952	0.0366*	0.1490	0.0121*
**laTL**	0.0267*	0.9432	0.3124	0.0894	0.3556	0.0413*
**raTL**	0.1057	0.9873	0.9367	0.0496*	0.0496*	0.1413
**lITG/ MTG**	0.0245*	0.9863	0.8921	0.0621	0.0621	0.0621
**rITG/ MTG**	0.0129*	0.9797	0.6615	0.0199*	0.0199*	0.0201*

Note. This table reports the precise *p*-values for the DCNN ResNet50 in Supplementary Figure 1 and corresponds to the data in Supplementary Table 8. We tested for the significance of the hypothesis model correlations against a null correlation using one-sided Wilcoxon signed-rank tests. Model comparisons were tested using paired, two-sided Wilcoxon signed rank tests. For the tests against zero, a black asterisk indicates Bonferroni-corrected significance considering the number of tests per ROI (N = 3; p < .017), a grey asterisk indicates uncorrected significance (p(unc.) < .001). For the model comparisons, an asterisk indicates FDR-corrected[4] significance for the model comparisons per ROI. OFA = occipital face area; pFFA = posterior fusiform face area aTL = anterior temporal lobe; ITG = inferior temporal gyrus; MTG = middle temporal gyrus.

Supplementary Table 10. Means and standard deviations for the main effects of the ROI analyses split-up by hemisphere.

VGG-Face

ROI	PE		Sharpening		Input		left		right	
	M	*SD*	*M*	*SD*	*M*	*SD*	*M*	*SD*	*M*	*SD*
**OFA**	0,0534	0.1143	0.0239	0.0852	0.0129	0.0866	0.0328	0.1001	0.0273	0.0949
**pFFA**	0.0333	0.1100	-0.0198	0.0915	-0.0076	0.0829	0.0065	0.0875	-0.0026	0.1072
**aTL**	0.0313	0.1182	-0.0069	0.0923	-0.0028	0.0929	0.0154	0.1126	-0.0010	0.0918
**ITG/ MTG**	0.0282	0.1118	-0.0079	0.0961	-0.0039	0.0944	0.0021	0.1054	0.0089	0.0987

VGG-16

ROI	PE		Sharpening		Input		left		right	
	M	*SD*	*M*	*SD*	*M*	*SD*	*M*	*SD*	*M*	*SD*
**OFA**	0.0529	0.1243	-0.0100	0.1168	0.0228	0.0930	0.0228	0.1117	0.0210	0.1179
**pFFA**	0.0907	0.1614	-0.0130	0.1235	0.0336	0.1411	0.0459	0.1452	0.0283	0.1518
**aTL**	0.0702	0.1255	-0.0214	0.1101	0.0245	0.1237	0.0273	0.1269	0.0215	0.1239
**ITG/ MTG**	0.0597	0.1547	-0.0092	0.1285	0.0263	0.1377	0.0179	0.1507	0.0333	0.1349

ResNet50

ROI	PE		Sharpening		Input		left		right	
	M	*SD*	*M*	*SD*	*M*	*SD*	*M*	*SD*	*M*	*SD*
**OFA**	0.0302	0.1558	-0.0233	0.1008	0.0061	0.0845	0.0083	0.1161	0.0003	0.1227
**pFFA**	0.0489	0.1370	-0.0352	0.1001	0.0037	0.0832	0.0117	0.0994	-0.0001	0.1271
**aTL**	0.0322	0.1307	-0.0299	0.0978	-0.0047	0.0907	0.0054	0.1170	-0.0071	0.1035
**ITG/ MTG**	0.0442	0.1280	-0.0395	0.1017	-0.0114	0.0921	-0.0051	0.1155	0.0006	0.1116

Note. PE = Prediction Error, ROI = region of interest, *M* = mean, *SD* = standard deviation, left = left hemisphere, right = right hemisphere, OFA = occipital face area, pFFA = posterior fusiform face area, aTL = anterior temporal lobe, ITG = inferior temporal gyrus, MTG = middle temporal gyrus.

Supplementary Table 11. Main effects and post-hoc tests for the ROI analyses split-up by hemisphere (VGG-Face).

Main effects and interaction of factors 'hemisphere' and 'model'

ROI	factor	*df*	*SS*	*MS*	*F*	*p*	η_p^2
**OFA**	hemisphere	1	3852.7481	3852.7481	0.9359	0.3344	0.0044
	model	2	39357.0233	19678.5116	4.9035	0.0083*	0.0446
	hemisphere:model	2	2557.5891	1278.7946	0.3097	0.7340	0.0029
**pFFA**	hemisphere	1	7370.7016	7370.7016	1.5714	0.2114	0.0074
	model	2	64890.3023	32445.1512	7.2968	0.0009*	0.0650
	hemisphere:model	2	4555.5891	2277.7946	0.4836	0.6173	0.0046
**aTL**	hemisphere	1	6763.7248	6763.7248	1.6550	0.1997	0.0078
	model	2	27796.4651	13898.2326	3.4311	0.0342*	0.0316
	hemisphere:model	2	8146.6589	4073.3295	1.0094	0.3662	0.0095
**MTG**	hemisphere	1	1937.3992	1937.3992	0.4857	0.4866	0.0023
	model	2	36569.1860	18284.5930	4.7405	0.0097*	0.0432
	hemisphere:model	2	4162.7674	2081.3837	0.5261	0.5917	0.0050

Post-hoc tests for main effect 'model'

ROI	contrast	*df*	*SE*	*t-ratio*	*p*
**OFA**	Input - PE	210	9.661	-3.0139	0.0081*
	Input - Sharpening	210	9.661	-0.7703	0.7216
	PE - Sharpening	210	9.661	2.2436	0.0664
**pFFA**	Input - PE	210	10.169	-2.8873	0.0119*
	Input - Sharpening	210	10.169	0.7227	0.7503
	PE - Sharpening	210	10.169	3.6100	0.0011*
**aTL**	Input - PE	210	9.706	-2.0127	0.1116
	Input - Sharpening	210	9.706	0.4457	0.8964
	PE - Sharpening	210	9.706	2.4584	0.0390*
**MTG**	Input - PE	210	9.471	-2.3511	0.0512
	Input - Sharpening	210	9.471	0.5463	0.8485
	PE - Sharpening	210	9.471	2.8975	0.0115*

Note. We tested the correlation values (Kendall's Tau A) for each region of interest (ROI) for main effects and interactions between the factors hemisphere (left, right) and model (PE, Sharpening, Sensory Input) using the non-parametric Aligned Rank Transform (ART)[11]. An asterisk indicates significance (p(unc.) < .05 for main effects and interaction, Tukey-corrected for post-hoc tests[12]). *Df* = degrees of freedom, *SS* = sum of squares, *MS* = mean square, *SE* = standard error of the mean, ROI = region of interest, OFA = occipital face area, pFFA = posterior fusiform face area, aTL = anterior temporal lobe, MTG = middle temporal lobe.

Supplementary Table 12. Main effects and post-hoc tests for the ROI analyses split-up by hemisphere (VGG-16).

Main effects and interaction of factors 'hemisphere' and 'model'

ROI	factor	*df*	*SS*	*MS*	*F*	*p*	η_p^2
**OFA**	hemisphere	1	240.3140	240.3140	0.0440	0.8340	0.0002
	model	2	56799.2326	28399.6163	5.4971	0.0047*	0.0497
	hemisphere:model	2	3831.9767	1915.9884	0.3514	0.7041	0.0033
**pFFA**	hemisphere	1	5242.5155	5242.5155	1.2246	0.2697	0.0058
	model	2	104916.3488	52458.1744	13.2052	0.0000*	0.1117
	hemisphere:model	2	2173.0465	1086.5233	0.2524	0.7772	0.0024
**aTL**	hemisphere	1	3132.5620	3132.5620	0.7089	0.4008	0.0034
	model	2	130361.0465	65180.5233	16.3647	0.0000*	0.1348
	hemisphere:model	2	5105.0930	2552.5465	0.5796	0.5610	0.0055
**MTG**	hemisphere	1	5152.7481	5152.7481	1.1194	0.2913	0.0053
	model	2	46720.4012	23360.2006	5.2176	0.0061*	0.0473
	hemisphere:model	2	1073.5814	536.7907	0.1162	0.8903	0.0011

Post-hoc tests for main effect 'model'

ROI	contrast	*df*	*SE*	*t-ratio*	*p*
**OFA**	Input - PE	210	10.9611	-1.5647	0.2633
	Input - Sharpening	210	10.9611	1.7493	0.1895
	PE - Sharpening	210	10.9611	3.3140	0.0031*
**pFFA**	Input - PE	210	9.6117	-2.5804	0.0283*
	Input - Sharpening	210	9.6117	2.5587	0.0300*
	PE - Sharpening	210	9.6117	5.1391	0.0000*
**aTL**	Input - PE	210	9.6243	-2.8151	0.0147*
	Input - Sharpening	210	9.6243	2.9057	0.0113*
	PE - Sharpening	210	9.6243	5.7207	0.0000*
**MTG**	Input - PE	210	10.2040	-1.6535	0.2257
	Input - Sharpening	210	10.2040	1.5766	0.2580
	PE - Sharpening	210	10.2040	3.2300	0.0041*

Note. We tested the correlation values (Kendall's Tau A) for each region of interest (ROI) for main effects and interactions between the factors hemisphere (left, right) and model (PE, Sharpening, Sensory Input) using the non-parametric Aligned Rank Transform (ART)[11]. An asterisk indicates significance (*p*(unc.) < .05 for main effects and interaction, Tukey-corrected for post-hoc tests[12]). *Df* = degrees of freedom, *SS* = sum of squares, *MS* = mean square, *SE* = standard error of the mean, ROI = region of interest, OFA = occipital face area, pFFA = posterior fusiform face area, aTL = anterior temporal lobe, MTG = middle temporal lobe.

Supplementary Table 13. Main effects and post-hoc tests for the ROI analyses split-up by hemisphere (ResNet50).

Main effects 'model' and 'hemisphere'

ROI	factor	*df*	*SS*	*MS*	*F*	*p*	η_p^2
**OFA**	hemisphere	1	1344.6550	1344.6550	0.2529	0.6156	0.0012
	model	2	43267.9767	21633.9884	4.2061	0.0162*	0.0385
	hemisphere:model	2	192.1473	96.0736	0.0180	0.9822	0.0002
**pFFA**	hemisphere	1	5260.5620	5260.5620	0.9338	0.3350	0.0044
	model	2	99196.3488	49598.1744	9.5595	0.0001*	0.0834
	hemisphere:model	2	2333.4961	1166.7481	0.2075	0.8128	0.0020
**aTL**	hemisphere	1	12909.3992	12909.3992	2.6021	0.1082	0.0122
	model	2	71736.9302	35868.4651	7.6288	0.0006*	0.0677
	hemisphere:model	2	2907.1705	1453.5853	0.2915	0.7475	0.0028
**MTG**	hemisphere	1	3760.5620	3760.5620	0.7321	0.3932	0.0035
	model	2	111785.9535	55892.9767	11.8647	0.0000*	0.1015
	hemisphere:model	2	269.3256	134.6628	0.0261	0.9742	0.0002

Post-hoc tests for main effect 'model'

ROI	contrast	*df*	*SE*	*t-ratio*	*p*
**OFA**	Input - PE	210	10.9369	-0.7995	0.7037
	Input - Sharpening	210	10.9369	2.0147	0.1112
	PE - Sharpening	210	10.9369	2.8142	0.0147*
**pFFA**	Input - PE	210	10.9845	-1.8493	0.1563
	Input - Sharpening	210	10.9845	2.5067	0.0344
	PE - Sharpening	210	10.9845	4.3560	0.0001*
**aTL**	Input - PE	210	10.4567	-2.2329	0.0681
	Input - Sharpening	210	10.4567	1.6591	0.2235
	PE - Sharpening	210	10.4567	3.8920	0.0004*
**MTG**	Input - PE	210	10.4669	-3.1972	0.0045*
	Input - Sharpening	210	10.4669	1.5842	0.2547
	PE - Sharpening	210	10.4669	4.7814	0.0000*

Note. We tested the correlation values (Kendall's Tau A) for each region of interest (ROI) for main effects and interactions between the factors hemisphere (left, right) and model (PE, Sharpening, Sensory Input) using the non-parametric Aligned Rank Transform (ART)[11]. An asterisk indicates significance (*p*(unc.) < .05 for main effects and interaction, Tukey-corrected for post-hoc tests[12]). *Df* = degrees of freedom, *SS* = sum of squares, *MS* = mean square, *SE* = standard error of the mean, ROI = region of interest, OFA = occipital face area, pFFA = posterior fusiform face area, aTL = anterior temporal lobe, MTG = middle temporal lobe.

Supplementary Table 14. Searchlight analysis for the hypothesis model Prediction Error (PE) (*p*(FWE) < .05) (VGG-Face, pool4).

peak *p*(FWE)	cluster equivk	peak T	peak equivZ	x,y,z {mm}	label
0.000	5841	6.61	5.44	52 -54 28	right angular gyrus
0.002		5.82	4.96	48 -58 -2	right middle temporal gyrus
0.004		5.50	4.75	32 -60 22	right lateral occipital cortex, superior division
0.000	10955	6.58	5.43	32 -8 48	right precentral gyrus
0.001		6.14	5.16	48 18 14	right inferior frontal gyrus, pars opercularis
0.002		5.83	4.96	12 22 40	right paracingulate gyrus
0.003	7916	5.67	4.86	-54 -54 46	left angular gyrus
0.003		5.61	4.82	-26 -86 26	left superior occipital gyrus
0.006		5.39	4.67	-44 -60 -4	left inferior temporal gyrus, temporooccipital part
0.010	157	5.17	4.52	-46 -24 -14	left middle temporal gyrus, posterior division
0.013	585	5.07	4.45	-44 14 8	left inferior frontal gyrus, pars opercularis
0.027		4.81	4.27	-34 26 26	left middle frontal gyrus
0.015	207	5.03	4.43	-42 26 48	left middle frontal gyrus
0.033		4.73	4.21	-30 10 56	
0.039		4.66	4.16	-30 16 50	
0.030	30	4.76	4.23	44 50 22	right middle frontal gyrus
0.032	65	4.74	4.22	56 -16 -16	right middle temporal gyrus, posterior division
0.042		4.63	4.14	60 -12 -30	
0.035	8	4.71	4.19	20 2 58	right superior frontal gyrus
0.037	14	4.68	4.17	-48 16 -14	left temporal pole
0.040	16	4.66	4.16	30 -28 -26	right fusiform gyrus
0.043	9	4.62	4.13	-38 58 6	left middle frontal gyrus
0.047	5	4.59	4.11	-4 56 12	left superior frontal gyrus medial segment
0.048	1	4.58	4.10	-18 10 -6	left putamen
0.048	3	4.58	4.10	30 14 2	right insular cortex
0.050	1	4.57	4.09	28 -22 -16	right hippocampus

Supplementary Table 15. Searchlight analysis for the hypothesis model Prediction Error (PE) (*p*(FWE) < .05) (VGG-Face, pool5).

peak *p*(FWE)	cluster equivk	peak T	peak equivZ	x,y,z {mm}	label
0.000	1498	6.95	5.64	30 -8 46	right precentral gyrus
0.004		5.54	4.77	34 -14 14	right posterior insula
0.031		4.79	4.26	20 -2 58	right superior frontal gyrus
0.002	1071	5.74	4.90	12 24 40	right paracingulate gyrus
0.011	463	5.19	4.54	-54 -54 44	left angular gyrus
0.031		4.79	4.26	-50 -64 32	
0.012	438	5.14	4.50	28 -66 28	right lateral occipital cortex, superior division
0.015		5.06	4.44	32 -60 22	
0.017		5.03	4.42	46 -54 28	right angular gyrus
0.014	264	5.08	4.46	48 16 14	right inferior frontal gyrus, pars opercularis
0.030		4.80	4.26	56 10 40	right precentral gyrus
0.040		4.69	4.18	50 6 24	
0.015	155	5.07	4.46	32 12 4	right insular cortex
0.016	54	5.04	4.43	-46 -24 -14	left middle temporal gyrus, posterior division
0.018	74	4.99	4.40	-28 8 54	left middle frontal gyrus
0.019	67	4.97	4.38	54 -16 -16	right middle temporal gyrus, posterior division
0.021	663	4.94	4.36	-28 -74 32	left lateral occipital cortex, superior division
0.029		4.82	4.28	-24 -90 34	left occipital pole
0.029		4.82	4.27	-34 -86 34	left middle occipital gyrus
0.028	39	4.83	4.28	-64 -58 -2	left middle temporal gyrus, temporooccipital part
0.034	7	4.76	4.23	-42 26 48	left middle frontal gyrus
0.037	28	4.73	4.21	-22 -62 -24	left cerebellum exterior
0.044	9	4.66	4.16	-40 14 12	left frontal operculum cortex
0.049	1	4.61	4.13	-10 -66 26	left precuneus
0.049	1	4.61	4.13	2 -14 36	right middle cingulate gyrus

Supplementary Table 16. Difference searchlight results for 'Prediction Error (PE) minus Sensory' (*p*(FWE) < .05) (VGG-Face, pool4 minus conv1_2).

peak *p*(FWE)	cluster equivk	peak T	peak equivZ	x,y,z {mm}	label
0.000	34547	6.92	5.62	50 4 42	right precentral gyrus
0.000		6.80	5.55	52 -46 36	right angular gyrus
0.000		6.73	5.51	-2 14 40	left middle cingulate gyrus
0.008	2080	5.30	4.61	0 -60 14	left precuneus
0.008		5.28	4.60	-6 -50 52	
0.009		5.23	4.56	-10 -48 22	left cingulate gyrus, posterior division
0.019	106	4.96	4.38	-46 -20 -14	left middle temporal gyrus, posterior division
0.024	93	4.88	4.32	-34 -54 -60	left cerebellum exterior
0.047	1	4.61	4.12	30 4 -32	right parahippocampal gyrus, anterior division
0.049	1	4.60	4.12	32 2 -30	right parahippocampal gyrus, anterior division
0.049	2	4.60	4.11	-38 54 4	left middle frontal gyrus

Supplementary Table 17. Difference searchlight results for 'Prediction Error (PE) minus Sensory' (*p*(FWE) < .05) (VGG-Face, pool5 minus conv1_2).

peak *p*(FWE)	cluster equivk	peak T	peak equivZ	x,y,z {mm}	label
0.000	2511	6.87	5.59	-2 12 42	left middle cingulate gyrus
0.009		5.27	4.59	2 -14 40	right middle cingulate gyrus
0.013		5.13	4.50	8 -16 46	right supplementary motor cortex
0.001	3221	6.18	5.18	32 -6 48	right precentral gyrus
0.001		5.94	5.03	42 10 -2	right anterior insula
0.002		5.84	4.97	52 4 40	
0.004	639	5.60	4.81	42 -46 22	right angular gyrus
0.045		4.66	4.16	42 -64 12	right lateral occipital cortex, inferior division
0.005	539	5.44	4.71	-24 -86 30	left superior occipital gyrus
0.008	349	5.33	4.63	-56 -52 46	left supramarginal gyrus, posterior division
0.012	466	5.17	4.52	34 24 48	right middle frontal gyrus
0.026		4.87	4.31	30 32 24	
0.013	1013	5.12	4.49	-46 14 18	left inferior frontal gyrus, pars opercularis
0.018		5.01	4.41	-34 24 50	left middle frontal gyrus
0.023		4.93	4.35	-38 26 32	
0.014	94	5.10	4.47	-48 16 -16	left temporal pole
0.015	176	5.08	4.46	54 -18 -16	right middle temporal gyrus, posterior division
0.016	112	5.04	4.43	-50 -22 -18	left middle temporal gyrus, posterior division
0.022	406	4.93	4.36	-44 -66 -2	left inferior occipital gyrus
0.032		4.80	4.26	-56 -72 6	left lateral occipital cortex, inferior division
0.037		4.74	4.22	-62 -64 -2	left middle temporal gyrus, temporooccipital part
0.024	135	4.90	4.34	-52 -68 30	left angular gyrus
0.046	2	4.65	4.15	-14 -62 -48	left cerebellum exterior

Supplementary Table 18. Searchlight analysis for the hypothesis model Sharpening (*p*(FWE) < .05) (VGG-Face, pool4).

peak *p*(FWE)	cluster equivk	peak T	peak equivZ	x,y,z {mm}	label
0.027	20	4.88	4.32	-6 68 14	left frontal pole
0.027	35	4.88	4.32	-52 -22 50	left postcentral gyrus
0.037	23	4.77	4.24	0 26 2	left subcallosal cortex

Supplementary Table 19. Difference searchlight results for 'Prediction Error (PE) minus Sharpening' (*p*(FWE) < .05) (VGG-Face, pool5) .

peak *p*(FWE)	cluster equivk	peak T	peak equivZ	x,y,z {mm}	label
0.003	380	5.65	4.84	12 22 42	right paracingulate gyrus
0.043		4.71	4.20	-4 12 44	left supplementary motor cortex
0.007	75	5.37	4.66	22 -6 56	right superior frontal gyrus
0.008	302	5.34	4.64	40 -46 22	right supramarginal gyrus, posterior division
0.016		5.09	4.47	32 -54 26	right parietal lobe
0.010	559	5.26	4.58	-16 -64 -22	left cerebellum exterior
0.013		5.15	4.51	-26 -66 -20	
0.015	148	5.12	4.48	-54 -50 46	left supramarginal gyrus
0.017	117	5.05	4.44	-48 10 8	left inferior frontal gyrus, pars opercularis
0.021	50	4.98	4.39	30 36 26	right middle frontal gyrus
0.025	37	4.91	4.34	-38 -56 -58	left cerebellum exterior
0.030	44	4.85	4.30	52 2 48	right precentral gyrus
0.040		4.74	4.22	48 -2 38	
0.033	19	4.81	4.27	54 -18 -16	right middle temporal gyrus
0.050	1	4.65	4.16	-60 8 8	left precentral gyrus

Supplementary Table 20. Searchlight analysis for the hypothesis model Prediction Error (PE) (*p*(FWE) < .05) (VGG-16, pool4).

peak *p*(FWE)	cluster equivk	peak T	peak equivZ	x,y,z {mm}	label
0.000	1988	6.69	5.49	34 -16 48	right precentral gyrus
0.004		5.53	4.77	34 -14 20	right insular cortex
0.001	2440	6.24	5.22	-40 28 48	left middle frontal gyrus
0.013		5.09	4.46	-48 16 -14	left temporal pole
0.017		4.98	4.39	-54 10 40	
0.001	2837	6.23	5.21	-50 -50 44	left supramarginal gyrus
0.006		5.36	4.65	-46 -58 -8	left inferior temporal gyrus, temporooccipital part
0.007		5.31	4.62	-56 -38 54	
0.001	1550	6.17	5.18	56 -48 48	right angular gyrus
0.001		5.90	5.01	50 -50 30	
0.002	1666	5.77	4.92	2 24 34	right paracingulate gyrus
0.003		5.66	4.85	2 18 40	
0.013		5.08	4.46	-2 38 52	left superior frontal gyrus medial segment
0.002	916	5.71	4.88	0 -50 18	left posterior cingulate gyrus
0.004	472	5.56	4.79	2 -14 42	right middle cingulate gyrus
0.031	26	4.76	4.23	34 36 18	right frontal pole
0.034	22	4.73	4.21	-4 -60 -20	cerebellar vermal lobules I-V
0.037	70	4.69	4.19	-22 -82 24	left superior occipital gyrus
0.040	18	4.67	4.17	-48 -8 -18	left superior temporal gyrus
0.048	2	4.60	4.11	36 -84 18	right middle occipital gyrus
0.049	3	4.59	4.11	50 8 42	right precentral gyrus
0.049	2	4.59	4.11	-24 -96 20	left occipital pole

Supplementary Table 21. Searchlight analysis for the hypothesis model Prediction Error (PE) (*p*(FWE) < .05) (VGG-16, pool5).

peak *p*(FWE)	cluster equivk	peak T	peak equivZ	x,y,z {mm}	label
0.000	10278	6.97	5.65	-36 28 52	left middle frontal gyrus
0.001		6.16	5.17	36 -8 48	right precentral gyrus
0.001		5.95	5.04	-6 40 52	left superior frontal gyrus
0.001	951	6.04	5.10	-4 -46 18	left posterior cingulate gyrus
0.041		4.64	4.15	-16 -56 34	left precuneus cortex
0.001	10506	5.91	5.01	-44 -58 16	left angular gyrus
0.002		5.81	4.95	-46 -58 4	left middle temporal gyrus
0.002		5.80	4.94	-50 -52 -38	left cerebellum exterior
0.002	1726	5.70	4.87	60 -54 38	right angular gyrus
0.004		5.52	4.76	58 -48 50	
0.045		4.60	4.11	54 -66 4	right inferior occipital gyrus
0.006	284	5.34	4.64	-2 -14 42	left middle cingulate gyrus
0.007	543	5.32	4.62	40 12 -6	right anterior insula
0.011	415	5.13	4.50	40 -84 12	right middle occipital gyrus
0.017	311	4.98	4.39	20 -40 -20	right cerebellum exterior
0.034		4.71	4.19	26 -28 -14	right hippocampus
0.017	151	4.98	4.39	6 -92 30	right occipital pole
0.020	82	4.92	4.35	32 26 52	right middle frontal gyrus
0.025	96	4.82	4.28	-68 -20 2	left superior temporal gyrus
0.029	80	4.77	4.24	-10 42 -10	left medial frontal cortex
0.033	36	4.72	4.20	30 -86 -36	right cerebellum exterior
0.042	22	4.63	4.14	38 -76 -18	right occipital fusiform gyrus
0.044	4	4.61	4.12	-18 10 -10	left putamen

Supplementary Table 22. Difference searchlight results for 'Prediction Error (PE) minus Sensory' (p(FWE) < .05) (VGG-16, pool4 minus conv1_2).

peak *p*(FWE)	cluster equivk	peak T	peak equivZ	x,y,z {mm}	label
0.007	523	5.34	4.64	0 24 36	paracingulate gyrus
0.007		5.33	4.63	0 14 42	
0.009	384	5.24	4.57	-36 30 40	left middle frontal gyrus
0.019	87	4.97	4.38	6 -14 54	right supplementary motor cortex
0.042		4.68	4.17	-2 -12 46	left middle cingulate gyrus
0.022	73	4.91	4.34	32 -16 22	right central opercular cortex
0.025	65	4.87	4.31	42 -10 52	right precentral gyrus
0.048	2	4.62	4.13	-44 18 26	left opercular part of the inferior frontal gyrus

Supplementary Table 23. Difference searchlight results for 'Prediction Error (PE) minus Sensory' (*p*(FWE) < .05) (VGG-16, pool5 minus conv1_2*).*

peak *p*(FWE)	cluster equivk	peak T	peak equivZ	x,y,z {mm}	label
0.005	172	5.42	4.69	32 -14 24	right parietal lobule
0.006	500	5.36	4.65	-32 30 52	left middle frontal gyrus
0.023		4.86	4.31	-38 34 36	
0.027		4.80	4.26	-44 40 14	
0.021	38	4.89	4.33	-6 40 56	left superior frontal gyrus
0.021	62	4.88	4.32	-28 20 -14	left posterior orbital gyrus
0.026	55	4.81	4.27	38 14 -4	right anterior insula
0.030	41	4.76	4.23	-44 -60 -4	left inferior temporal gyrus, temporooccipital part
0.030	18	4.75	4.23	-56 -60 -34	left cerebellum
0.032	10	4.73	4.21	14 66 24	right frontal pole
0.039	17	4.66	4.16	-46 50 0	left middle frontal gyrus
0.043	11	4.62	4.13	2 16 42	right paracingulate gyrus
0.044	12	4.61	4.13	40 -86 16	right middle occipital gyrus
0.045	7	4.60	4.12	-50 8 30	left precentral gyrus
0.047	1	4.59	4.11	-42 -58 20	left angular gyrus
0.047	3	4.58	4.10	36 32 34	right middle frontal gyrus

Supplementary Table 24. Difference searchlight results for ‘Prediction Error (PE) minus Sharpening’ (*p*(FWE) < .05) (VGG-16, pool4).

peak *p*(FWE)	cluster equivk	peak T	peak equivZ	x,y,z {mm}	label
0.002	658	5.85	4.97	-38 28 50	left middle frontal gyrus
0.002	608	5.70	4.88	36 -14 46	right precentral gyrus
0.013		5.11	4.48	32 -16 30	right parietal lobule
0.034		4.74	4.21	34 -16 22	right insular cortex
0.007	541	5.30	4.61	12 22 38	right paracingulate gyrus
0.009		5.25	4.58	2 16 42	
0.010		5.21	4.55	2 24 34	
0.022	137	4.91	4.34	-42 16 -10	left insular cortex
0.033	34	4.75	4.23	8 -14 50	right precentral gyrus
0.034	118	4.74	4.22	-44 36 12	left triangular part of the inferior frontal gyrus
0.038	19	4.69	4.18	36 -86 18	right middle occipital gyrus
0.047	2	4.61	4.12	-52 -52 42	left supramarginal gyrus

Supplementary Table 25. Difference searchlight results for 'Prediction Error (PE) minus Sharpening' (p(FWE) < .05) (VGG-16, pool5).

peak *p*(FWE)	cluster equivk	peak T	peak equivZ	x,y,z {mm}	label
0.000	1115	6.50	5.38	-34 28 52	left middle frontal gyrus
0.006		5.35	4.64	-42 26 36	
0.008	87	5.24	4.57	-4 42 54	left superior frontal gyrus
0.009	103	5.22	4.56	-44 -20 -38	left inferior temporal gyrus
0.010	546	5.16	4.52	-46 40 10	left triangular part of the inferior frontal gyrus
0.016	94	4.99	4.40	18 64 24	right frontal pole
0.035	25	4.70	4.19	34 -86 16	right middle occipital gyrus
0.039	20	4.66	4.16	-2 28 36	left superior frontal gyrus medial segment
0.043	8	4.61	4.13	-2 -48 18	left posterior cingulate gyrus
0.047	3	4.58	4.10	-30 -60 30	left lateral occipital cortex, superior division
0.049	2	4.57	4.09	-50 -50 -38	left cerebellum exterior

Supplementary Table 26. Searchlight analysis for the hypothesis PE model, *p*(unc.) < .001 + cluster-corrected *p*(FWE) < .05, *k* > 10 voxel) (ResNet50, res5b_branch2b).

cluster *p*(FWE)	cluster equivk	peak *p*(FWE)	peak T	peak equivZ	x,y,z {mm}	label
0.000	15925	0.002	5.80	4.94	-38 28 50	left middle frontal gyrus
		0.023	4.87	4.31	0 26 34	paracingulate gyrus
		0.049	4.57	4.09	38 -12 50	right precentral gyrus
0.000	6163	0.033	4.73	4.21	-50 -48 -38	left cerebellum exterior
		0.119	4.21	3.82	-42 -58 -6	left inferior temporal gyrus
		0.155	4.09	3.73	-44 -46 52	left supramarginal gyrus
0.002	3876	0.035	4.70	4.19	56 -48 48	right angular gyrus
		0.045	4.61	4.12	50 -48 34	
		0.165	4.06	3.71	28 -66 28	right lateral occipital cortex, superior division
0.039	1392	0.059	4.50	4.04	8 -46 26	right cingulate gyrus, posterior division

Supplementary Table 27. Difference searchlight results for 'Prediction Error (PE) minus Sensory', *p*(unc.) < .001 + cluster-corrected *p*(FWE) < .05, *k* > 10 voxel (ResNet50, res5b_branch2b).

cluster *p*(FWE)	cluster equivk	peak *p*(FWE)	peak T	peak equivZ	x,y,z {mm}	label
0.001	4705	0.052	4.57	4.09	-38 30 36	left middle frontal gyrus
		0.069	4.46	4.01	-34 28 48	
		0.096	4.32	3.91	-48 50 0	left frontal pole
0.009	2495	0.126	4.21	3.82	42 26 36	right middle frontal gyrus
		0.190	4.03	3.68	38 18 44	
		0.215	3.97	3.64	48 -8 52	right precentral gyrus

Supplementary Table 28. Difference searchlight results for 'Prediction Error (PE) minus Sharpening', *p*(unc.) < .001 + cluster-corrected *p*(FWE) < .05, *k* > 10 voxel (ResNet50, res5b_branch2b).

cluster *p*(FWE)	cluster equivk	peak *p*(FWE)	peak T	peak equivZ	x,y,z {mm}	label
0.000	17100	0.000	6.30	5.25	-38 28 50	left middle frontal gyrus
		0.010	5.17	4.52	6 -14 50	right supplementary motor cortex
		0.014	5.06	4.45	-2 26 34	left superior frontal gyrus medial segment
0.004	3231	0.044	4.61	4.12	56 -44 52	right supramarginal gyrus
		0.092	4.32	3.91	34 -86 18	right middle occipital gyrus
		0.167	4.06	3.71	58 -62 26	right angular gyrus
0.002	3722	0.079	4.38	3.96	-44 -58 -6	left inferior temporal gyrus, temporooccipital part
		0.206	3.97	3.64	-28 -58 28	left angular gyrus
		0.238	3.90	3.58	-48 -54 -22	left inferior temporal gyrus

Supplementary Table 29. Localizer ‘faces > scenes’, *p*(unc.) < .001 + cluster-corrected *p*(FWE) < .05, *k* > 10 voxel.

cluster *p*(FWE)	cluster equivk	peak *p*(FWE)	peak T	peak equivZ	x,y,z {mm}	label
0.000	6300	0.000	14.14	Inf	48 -62 2	right middle temporal gyrus
		0.000	12.33	Inf	52 -70 -2	
		0.000	10.40	7.28	42 -70 -8	
0.000	2887	0.000	10.75	7.41	-46 -72 4	left inferior occipital gyrus
		0.000	9.75	7.01	-50 -76 -6	
		0.000	7.89	6.14	-44 -52 -20	
0.001	444	0.007	6.17	5.18	-8 -76 -44	left cerebellum exterior
		0.151	5.05	4.44	-22 -78 -56	
0.000	820	0.026	5.72	4.89	52 8 32	right precentral gyrus
		0.227	4.88	4.32	54 10 16	
		0.494	4.50	4.05	48 2 42	
0.000	730	0.034	5.62	4.82	-38 -36 40	left supramarginal gyrus
		0.205	4.93	4.35	-62 -26 46	
		0.842	4.10	3.74	-54 -26 34	
0.019	262	0.042	5.54	4.78	44 36 8	right triangular part of the inferior frontal gyrus

Supplementary References

1. He, K., Zhang, X., Ren, S. & Sun, J. Deep Residual Learning for Image Recognition. in *2016 IEEE Conference on Computer Vision and Pattern Recognition (CVPR)* 770–778 (2016). doi:10.1109/CVPR.2016.90.
2. Ratan Murty, N. A., Bashivan, P., Abate, A., DiCarlo, J. J. & Kanwisher, N. Computational models of category-selective brain regions enable high-throughput tests of selectivity. *Nat. Commun.* **12**, 5540 (2021).
3. Morey, R. D. Confidence Intervals from Normalized Data: A correction to Cousineau (2005). *Tutor. Quant. Methods Psychol.* **4**, 61–64 (2008).
4. Benjamini, Y. & Hochberg, Y. Controlling the False Discovery Rate: A Practical and Powerful Approach to Multiple Testing. *J. R. Stat. Soc. Ser. B Methodol.* **57**, 289–300 (1995).
5. Nili, H. *et al.* A Toolbox for Representational Similarity Analysis. *PLOS Comput. Biol.* **10**, e1003553 (2014).
6. Dobs, K., Isik, L., Pantazis, D. & Kanwisher, N. How face perception unfolds over time. *Nat. Commun.* **10**, 1–10 (2019).
7. Parkhi, O. M., Vedaldi, A. & Zisserman, A. Deep Face Recognition. in *Procedings of the British Machine Vision Conference 2015* 41.1-41.12 (British Machine Vision Association, Swansea, 2015). doi:10.5244/C.29.41.
8. Blank, H., Kiebel, S. J. & von Kriegstein, K. How the human brain exchanges information across sensory modalities to recognize other people: Information Across Sensory Modalities. *Hum. Brain Mapp.* **36**, 324–339 (2015).
9. Hebart, M. N., Görgen, K. & Haynes, J.-D. The Decoding Toolbox (TDT): a versatile software package for multivariate analyses of functional imaging data. *Front. Neuroinformatics* **8**, (2015).
10. Chang, C.-C. & Lin, C.-J. LIBSVM: A library for support vector machines. *ACM Trans. Intell. Syst. Technol.* **2**, 27:1-27:27 (2011).

11. Wobbrock, J. O., Findlater, L., Gergle, D. & Higgins, J. J. The aligned rank transform for nonparametric factorial analyses using only anova procedures. in *Proceedings of the SIGCHI Conference on Human Factors in Computing Systems* 143–146 (ACM, Vancouver BC Canada, 2011). doi:10.1145/1978942.1978963.

12. Elkin, L. A., Kay, M., Higgins, J. J. & Wobbrock, J. O. An Aligned Rank Transform Procedure for Multifactor Contrast Tests. in *The 34th Annual ACM Symposium on User Interface Software and Technology* 754–768 (ACM, Virtual Event USA, 2021). doi:10.1145/3472749.3474784.
