# Supplementary material for: Prediction error processing and sharpening of expected information across the face-processing hierarchy
Source: Nat Commun. 2024 Apr 22;15:3407. doi: 10.1038/s41467-024-47749-9 (PMC11035707; doi:10.1038/s41467-024-47749-9)
Supplement: Supplementary file 3 — Reporting Summary [file 41467_2024_47749_MOESM3_ESM.pdf]

nature portfolio

Corresponding author(s): Garlichs, A. & Blank, H.

Last updated by author(s): Mar 22, 2024

Reporting Summary

Nature Portfolio wishes to improve the reproducibility of the work that we publish. This form provides structure for consistency and transparency in reporting. For further information on Nature Portfolio policies, see our Editorial Policies and the Editorial Policy Checklist.

Statistics

For all statistical analyses, confirm that the following items are present in the figure legend, table legend, main text, or Methods section.

n/a	Confirmed	
☐	☒	The exact sample size (*n*) for each experimental group/condition, given as a discrete number and unit of measurement
☐	☒	A statement on whether measurements were taken from distinct samples or whether the same sample was measured repeatedly
☐	☒	The statistical test(s) used AND whether they are one- or two-sided<br>*Only common tests should be described solely by name; describe more complex techniques in the Methods section.*
☒	☐	A description of all covariates tested
☐	☒	A description of any assumptions or corrections, such as tests of normality and adjustment for multiple comparisons
☐	☒	A full description of the statistical parameters including central tendency (e.g. means) or other basic estimates (e.g. regression coefficient) AND variation (e.g. standard deviation) or associated estimates of uncertainty (e.g. confidence intervals)
☐	☒	For null hypothesis testing, the test statistic (e.g. *F*, *t*, *r*) with confidence intervals, effect sizes, degrees of freedom and *P* value noted<br>*Give P values as exact values whenever suitable.*
☒	☐	For Bayesian analysis, information on the choice of priors and Markov chain Monte Carlo settings
☒	☐	For hierarchical and complex designs, identification of the appropriate level for tests and full reporting of outcomes
☐	☒	Estimates of effect sizes (e.g. Cohen's *d*, Pearson's *r*), indicating how they were calculated

Our web collection on statistics for biologists contains articles on many of the points above.

Software and code

Policy information about availability of computer code

Data collection	We programmed our experiments using MATLAB R2020b ([https://de.mathworks.com]) and Psychtoolbox (v3.0.18; [www.psychtoolbox.org]). For stimulus presentation and data collection, we used different MATLAB and Psychtoolbox versions (MATLAB: R2016b, R2020b; Psychtoolbox: v3.014, v3.0.17, v3.0.18).
Data analysis	Custom code for behavioural (MATLAB, R) and multivariate analyses (MATLAB, Python) is available at the Open Science Framework (OSF) ([https://osf.io/765jx/]). For our behavioural data analyses, we used MATLAB R2020b and R/RStudio (R v4.2.0, [https://www.r-project.org]; Rstudio v2022.02.2; [https://posit.co]). For our univariate fMRI data analyses, we used SPM12 ([https://www.fil.ion.ucl.ac.uk/spm/software/spm12]). For our multivariate RSA, we used the RSAtoolbox (Nili et al., 2014; v0.0.4; [https://github.com/rsagroup/rsatoolbox]) in Python 3.9.12. For neuroanatomical labelling, we used the Neuromorphmetrics atlas (Neuromorphometrics, Inc.) implemented in SPM12 as well as the Harvard-Oxford Cortical Structural Atlas and the Harvard-Oxford Subcortical Structural Atlas in FSLeyes (v0.24.3). For visualisation, we used MRIcroGL (v1.2.20220720; [https://www.nitrc.org/projects/mricrogl]). For our non-parametric analysis of variance for the RSA split-up by hemisphere, we used the ARTool-package (Wobbrock et al., 2011; Elkin et al., 2021; v0.11.1; [https://cran.r-project.org/web/packages/ARTool/index.html]) in RStudio. For our multivariate classification analyses, we used The Decoding Toolbox (Hebart et al., 2015; v3.999F; [https://sites.google.com/site/tdtdecodingtoolbox/]) in MATLAB R2020b.

For manuscripts utilizing custom algorithms or software that are central to the research but not yet described in published literature, software must be made available to editors and reviewers. We strongly encourage code deposition in a community repository (e.g. GitHub). See the Nature Portfolio guidelines for submitting code & software for further information.

Data

Policy information about availability of data

All manuscripts must include a data availability statement. This statement should provide the following information, where applicable:

- Accession codes, unique identifiers, or web links for publicly available datasets
- A description of any restrictions on data availability
- For clinical datasets or third party data, please ensure that the statement adheres to our policy

The face stimuli used in this study were created with FaceGen Modeller Core 3.22 (Singular Inversion; [https://facegen.com]) and are available at OSF ([https://osf.io/765jx/]).
The scene images used in this study were taken from the SUN database (Xiao et al., 2010; [https://groups.csail.mit.edu/vision/SUN/hierarchy.html]) and the indoor scene database (Quattoni & Torralba, 2009; [https://web.mit.edu/torralba/www/indoor.html]) and are available at [[https://osf.io/765jx/]. The exemplary scene image in Figure 1 is in public domain and available at [https://commons.wikimedia.org].
The VGG-Face model (Parkhi et al., 2015) used in this study is available at [www.robots.ox.ac.uk/~vgg/software/vgg_face/].
The VGG-16 (Simonyan & Zisserman, 2015) and ResNet50 (He et al., 2016) models used in this study, pre-trained on the ImageNet dataset, are available via MATLAB ([https://de.mathworks.com/help/deeplearning/ref/vgg16.html]; [https://de.mathworks.com/help/deeplearning/ref/resnet50.html]).
Source data are provided with this paper. The raw behavioural and fMRI data generated in this study are available from the authors upon reasonable request.

Research involving human participants, their data, or biological material

Policy information about studies with human participants or human data. See also policy information about sex, gender (identity/presentation), and sexual orientation and race, ethnicity and racism.

Reporting on sex and gender	We preregistered to measure 50 participants. Seven participants were excluded from final data analyses: one due to technical issues, one due to anatomical anomalies, one due to extensive head movements, three did not take part in all study appointments, and one was identified as an outlier in the behavioural analysis. Our final data analyses are based on 43 participants (m = 21, f = 22). Gender was determined based on self-reporting (options: 'male', 'female', 'diverse', 'not listed'). The sample was balanced for gender.
Reporting on race, ethnicity, or other socially relevant groupings	We did not register information about race, ethnicity, or other socially relevant groupings and did not control for these in our analyses.
Population characteristics	In the final sample, 43 right-handed participants (m = 21, f = 22) with mean age 24.37 years (SD = 3.61) were analysed. Inclusion criteria for study participation were age (18-35 years old), very good German skills, and right-handedness. Exclusion criteria were current or past neurological or psychiatric disorders, visual or hearing impairments, glasses, regular medication or substance addiction, MR-incompability, or difficulties in face recognition.
Recruitment	Participants were recruited via the website Stellenwerk ([https://www.stellenwerk.de/hamburg/]) or contacted if they had taken part in former studies of the Department of Systems Neurosciences at the University Medical Center in Hamburg-Eppendorf (UKE), Germany, and agreed to be informed about future studies. Compensation for participation was 55 €. We followed standard procedures in the field to recruit participants that should not induce any specific biases in our results.
Ethics oversight	All experimental procedures were approved by the Ethics Committee of the Chamber of Physicians in Hamburg and participants provided written informed consent.

Note that full information on the approval of the study protocol must also be provided in the manuscript.

Field-specific reporting

Please select the one below that is the best fit for your research. If you are not sure, read the appropriate sections before making your selection.

☒ Life sciences ☐ Behavioural & social sciences ☐ Ecological, evolutionary & environmental sciences

For a reference copy of the document with all sections, see nature.com/documents/nr-reporting-summary-flat.pdf

Life sciences study design

All studies must disclose on these points even when the disclosure is negative.

Sample size	We preregistered to measure 50 participants ([https://osf.io/sd54e]). The sample size of 50 for this study has been granted as part of an Emmy Noether funding from the DFG. This sample size has been shown to produce reliable fMRI results (Turner et al., 2018) and a sample size of 40 captured population-level effects of interest in a recent meta-analysis (Geuter et al., 2018). Data loss due to excessive movement, etc., was estimated at 10%.
Data exclusions	Seven participants were excluded from final data analyses: one due to technical issues, one due to anatomical anomalies, one due to extensive head movements, three did not take part in all study appointments, and one was identified as an outlier in the behavioural analysis. The excluded participant had a standardized z-value of -4.16, i.e., performing worse than 99.9% of the participants (based on the mean

accuracies of the match, mismatch, partial, catch, neutral, and neutral catch conditions).

Replication	In order to ensure reproducibility of our experimental findings, we documented the experimental procedures as well as statistical analyses in the Methods section and the Supplementary Methods. Furthermore, our custom code for behavioural (MATLAB, R) and multivariate analyses (MATLAB, Python) are available for download from OSF ([https://osf.io/765jx/]).
Randomization	All participants completed the whole experiment, no assignment to different groups was made.<br>The pairing of scenes and face images was counter-balanced across participants. In total, there were five different scene-face associations. The fMRI experiment contained four blocks. The ratio of trials per condition (match, partial, mismatch, catch, neutral, neutral catch, null events) was identical in all four blocks, but the trial order was pseudo-randomized within each block. The same identity (100% face, or morph image) was restricted to consecutively appear four times at the maximum. It was also controlled that only two null event trials could appear consecutively after each other to avoid too long periods of fixation crosses and, therefore, potential attentional losses by the participants.
Blinding	Blinding was not relevant for our study because we had one experimental group.

Reporting for specific materials, systems and methods

We require information from authors about some types of materials, experimental systems and methods used in many studies. Here, indicate whether each material, system or method listed is relevant to your study. If you are not sure if a list item applies to your research, read the appropriate section before selecting a response.

Materials & experimental systems

n/a	Involved in the study
☒	☐ Antibodies
☒	☐ Eukaryotic cell lines
☒	☐ Palaeontology and archaeology
☒	☐ Animals and other organisms
☒	☐ Clinical data
☒	☐ Dual use research of concern
☒	☐ Plants

Methods

n/a	Involved in the study
☒	☐ ChIP-seq
☒	☐ Flow cytometry
☐	☒ MRI-based neuroimaging

Plants

Seed stocks	*Report on the source of all seed stocks or other plant material used. If applicable, state the seed stock centre and catalogue number. If plant specimens were collected from the field, describe the collection location, date and sampling procedures.*
Novel plant genotypes	*Describe the methods by which all novel plant genotypes were produced. This includes those generated by transgenic approaches, gene editing, chemical/radiation-based mutagenesis and hybridization. For transgenic lines, describe the transformation method, the number of independent lines analyzed and the generation upon which experiments were performed. For gene-edited lines, describe the editor used, the endogenous sequence targeted for editing, the targeting guide RNA sequence (if applicable) and how the editor was applied.*
Authentication	*Describe any authentication procedures for each seed stock used or novel genotype generated. Describe any experiments used to assess the effect of a mutation and, where applicable, how potential secondary effects (e.g. second site T-DNA insertions, mosiacism, off-target gene editing) were examined.*

Magnetic resonance imaging

Experimental design

Design type	The experiment was an event-related design. The functional localizer was a block-design (faces, scenes, neutral fixation cross).
Design specifications	The fMRI experiment was divided into four blocks. Each block consisted of 107 experimental trials (16 match, 48 partial, 12 mismatch, 12 catch, 16 neutral, 3 neutral catch) and 36 null events. Each trial lasted 5300 ms and consisted of a jittered inter-trial interval (2-4 s, mean: 3 s), a scene image (400 ms), a jittered ISI (700-900 ms, mean: 800 ms), a face image (100 ms), and a response window (1000 ms). Each block lasted ~12 min, the whole experiment took ~53 min.
Behavioral performance measures	Since values of (1) perceived face identity in face morphs, (2) reaction times (RT), as well as (3) accuracies were not normally distributed (Kolmogorow-Smirnow tests, all p < .001), non-parametric tests were used for the analyses instead of the preregistered parametric tests.<br><br>(1) We measured perceived face identity. In each trial, the participants' task was to indicate which identity they recognized in the noisy face image/morph by pressing the respective button. For each participant, a difference score was calculated for each face pair to indicate how likely the participant answered in favour of the prior. The mean of the difference scores of all scene and morph combinations was calculated to obtain an individual index for an assimilation and/or contrastive effect. These scores were transformed into percentages: Values above 50% indicated that a

participant responded more often in favour of the expected face identity in a face morph (assimilation effect). Values below 50% were indicative of a contrastive effect. On the group level, the alternative hypothesis was tested that the participants' scores significantly differed from 50% (no prior-effect) using a two-sided Wilcoxon signed rank test. Wilcoxon's r was calculated as a measurement of effect size.

(2) We measured RTs (ms) for the time point of a button press after face onset. Firstly, we calculated a Friedman test with the factor condition (match, mismatch, neutral, partial) based on mean RTs for each condition per participant. Kendall's W was calculated as a measurement of effect size. Post-hoc paired t-tests between the average ranks of the different conditions were performed using Tukey's honestly significant difference (HSD) test for multiple comparisons. Secondly, we investigated whether the RTs to the morphed faces in partial trials depended on the response given by the participants. Therefore, partial trials were split into trials with prior-confirming responses (assimilation effect) and trials with responses favouring the other identity contained in a morph (contrastive effect) and tested with a paired two-sided Wilcoxon signed rank test. Lastly, we tested whether RTs in trials with prior-confirming responses differed from RTs in the match condition using a two-sided Wilcoxon signed rank test. Wilcoxon's r was calculated as a measurement of effect size.

(3) For controlling whether participants were attentive and correctly performed the task, a Friedman test with the within-subject factor condition (match, mismatch, catch, neutral, neutral catch) and accuracy (%) as the dependent variable was calculated. Match and mismatch trials were classified as correct if the presented (unmorphed) face was correctly identified in time. In catch trials, participants had to correctly answer the question mark by indicating which person they expected based on the preceding scene. Neutral trials were correct if participants pressed the button with the left thumb irrespective of which face was presented and were correctly answered if they pressed the button with the left index finger to indicate that all persons were equally likely to be expected. Note that for the conditions mentioned, no morphs were presented. Kendall's W was calculated as a measurement of effect size. For post-hoc tests, Tukey's HSD test for multiple comparisons was used.

Acquisition

Imaging type(s): functional, structural

Field strength: 3T

Sequence & imaging parameters: Functional data were obtained using a multiband echo-planar imaging sequence (repetition time (TR) = 0.961s, echo time (TE) = 30ms, flip angle = 55°, field of view (FoV) = 224mm, multi-band mode, number of bands: 3). Each volume of the experimental data contained 45 slices (voxel size 2 × 2 × 2mm plus 0.5 mm gap) and were obtained in descending order.

An additional structural image (magnetization prepared rapid acquisition gradient echo (MPRAGE)) was acquired for functional preprocessing and anatomical overlay (TR = 7.1 ms, TE = 2.98 ms, flip angle = 9°, FoV = 256 mm, 240 slices, voxel size 1 × 1 × 1mm, ascending order).

A fieldmap was acquired for field inhomogeneity corrections (TR = 495 ms, TE1 = 5.51 ms, TE2 = 7.97 ms, flip angle = 40°, FoV = 224 mm, 45 slices (voxel size 3 x 3 x 2 mm plus 0.5 mm gap). The slices were obtained in an interleaved order.

The protocols with scanning parameters are available here: [https://osf.io/765jx/].

Area of acquisition: whole-brain

Diffusion MRI: ☐ Used ☒ Not used

Preprocessing

Preprocessing software: Structural and functional data were analysed using SPM12 and custom scripts in MATLAB. First, the functional images of all functional runs were realigned to the mean functional image. We then applied field mapping distortion correction to the functional volumes to correct for geometric distortions in EPI caused by magnetic field inhomogeneity (with the FieldMap toolbox). The individual structural T1 image was co-registered to the mean, distortion-corrected functional image. The functional images were spatially normalised to MNI space. For the univariate analysis, the functional images were additionally smoothed with a 8-mm full-width at half maximum isotropic Gaussian kernel.

Normalization: Data were normalized using a 4th Degree B-Spline Interpolation.

Normalization template: Data were normalized based on SPM12's implemented tissue probability maps (TPM.nii).

Noise and artifact removal: We applied field mapping distortion correction to the functional volumes to correct for geometric distortions in EPI caused by magnetic field inhomogeneity (with the FieldMap toolbox).
Raw motion parameters (three translations and three rotations) were included as regressors of nuisance on the first-level.

Volume censoring: none

Statistical modeling & inference

Model type and settings: Univariate analyses:
Data of the four functional runs were analysed using the general linear model (GLM) with a 128 s high pass filter. We applied

SPM's alternative pre-whitening method to account for autocorrelation, FAST, which has been suggested to perform better than SPM's default (Olszowy et al., 2019). First-level models were estimated based on individual normalized, smoothed functional images.

Multivariate analyses/RSA (Kriegeskorte, 2008; Nili et al., 2014):
First-level models were estimated based on individual native, realigned functional images.

Effect(s) tested

Main experiment:
On the second level, we computed the 1) 'mismatch > match' and the 2) 'unexpected > expected' contrasts (one-sample t-tests).

Functional localizer:
On the second level, we computed the 'faces > scenes' contrast (one-sample t-test).

Multivariate analyses:
RSA (Kriegeskorte, 2008; Nili et al., 2014):
Our neural representational dissimilarity matrices (RDM) were based on the T-images of the neutral and partial conditions. We calculated Kendall's Tau A correlations between the hypothesised and the neural dissimilarity structure which have been recommended for hypothesis RDMs that predict tied ranks (Nili et al., 2014). Since correlation values for the different models (Prediction Error (PE), Sharpening, Sensory Input) and ROIs were not normally distributed (Kolmogorow-Smirnow tests, p < .001), we used non-parametric tests to test for significance. In our multivariate ROI analyses, the correlations for each hypothesis model were tested against zero using one-sided Wilcoxon signed rank tests. For the model comparisons, we used paired, two-sided Wilcoxon signed rank tests. For comparing left to right hemispheric correlations, we performed analyses of variances using ARTool (Wobbrock et al., 2011) as well as post-hoc pairwise comparisons (Elkin et al., 2021). Lastly, we report whole-brain searchlight analysis results for our different hypothesis models based on individual Fisher's z-transformed correlation maps using one-sample t-tests.

Classification analysis:
In addition to the preregistered RSA, we conducted a simpler multivariate classification approach without model-based hypotheses RDMs using The Decoding Toolbox (Hebart et al., 2015) using L2-norm support vector machines (SVM) from the library LIBSVM (Chang & Lin, 2011). We tested whether a 50/50 morphed face is classified as the expected or unexpected face identity in our ROIs. As classification scores were not normally distributed (Kolmogorov-Smirnow tests, all p < .001), we used two-sided Wilcoxon signed rank tests for all classification analyses.

Specify type of analysis: ☐ Whole brain ☐ ROI-based ☒ Both

Anatomical location(s)

We obtained our regions of interest (ROI) (lOFA, rOFA, lpFFA, rpFFA) from our functional localizer contrast 'faces > scenes' (p(unc.) < .001, cluster-corrected p(FWE) < .05). This yielded huge lateral clusters in the temporal cortex that were not seperable into OFA and FFA. We overlayed our activation clusters with the OFA and pFFA clusters by Zhen et al. (2015) to obtain our final OFA and pFFA ROIs.

The aTL ROIs were obtained by the functional localizer contrast 'faces > scenes' (p(unc.) < .01).

The ITG/MTG ROIs were obtained by the main experiment contrast mismatch > match' (p(unc.) < .001, cluster-corrected p(FWE) < .05). For the right hemispheric ROI, to have a comparable size, the threshold was lowered to p(unc.) < .01.

Statistic type for inference

(See Eklund et al. 2016)

whole-brain analyses: cluster-wise; ROI analyses: voxel-wise

Correction

Univariate analyses:
For the whole-brain analyses, we report cluster activations (p(FWE) < .05 with cluster inducing threshold of p < .001). For the small-volume corrected analyses of our ROIs (OFA, pFFA, aTL), we report peak activations (p(FWEsmall-volume corrected < .05).

Multivariate analyses:
For our multivariate ROI analyses, the correlations for each hypothesis model (PE, Sharpening, Sensory Input) were tested against zero. Significance was evaluated by Bonferroni-correcting for the number of tests per ROI (VGG-Face and VGG-16: N = 6 (3 models x 2 DCNNs); ResNet50: N = 3). For the model comparisons, significance was inferred by FDR-correcting (Benjamini & Hochberg, 1995) the p-values for the model comparisons per ROI (all model comparisons within each DCNN and main model comparisons across the DCNNs, i.e., PE vs. PE, Sharpening vs. Sharpening, and Sensory Input vs. Sensory Input). For the comparison of left and right hemispheric correlations using ARTool (Wobbrock et al., 2011), significance for main effects and interactions was evaluated by p(unc.) < .05, for post-hoc pairwise comparisons by p < .05, Tukey-corrected (Elkin et al., 2021). Furthermore, we report whole-brain searchlight analysis results for our different hypothesis models (p(FWE) < .05; ResNet: p(FWE) < .05 (cluster-corrected), cluster-inducing threshold of p(unc.) < .001). Finally, we report exploratory classification ROI analyses of the face morphs as the expected or unexpected face identity at p(unc.) < .05.

Models & analysis

n/a	Involved in the study
☒	☐ Functional and/or effective connectivity
☒	☐ Graph analysis
☐	☒ Multivariate modeling or predictive analysis

Multivariate modeling and predictive analysis

RSA involves defining theoretical dissimilarity matrices (i.e., hypothesis RDMs) between experimental conditions and comparing them to neural dissimilarity matrices (i.e., neural RDMs) based on the measured brain activation. In this study, we defined three hypothesis model RDMs (PE, Sharpening, Sensory Input) to test how presented faces are represented depending on prior context. All three hypothesis RDMs were based on the neural network activations of the deep neural network VGG-Face (Parkhi et al., 2015). We used the fixed models option of the RSAtoolbox, but each participant had their own individually behaviour-weighted hypothesis models. For each ROI, we obtained one Kendall's Tau A correlation coefficient for each participant for each hypothesis RDM. To explore the specificity of presented and predicted face representations within the whole brain, a multivariate searchlight approach was applied and the same correlation analyses as in the ROI approach were computed.

In addition to the preregistered RSA, we conducted a simpler multivariate classification approach without model-based hypotheses RDMs to evaluate whether a face morph is classified as the expected of unexpected face identity in our ROIs.
